# Three New Secondary Metabolites from *Colletotrichum boninense*, an Endophytic Fungus in *Polygonum multiflorum*

**DOI:** 10.3390/molecules31213855

**Published:** 2026-10-26

**Authors:** Tangqian Wu, Chengying Luo, Yanning Nong, Yazhou Zhang, Yuan Zhou

**Affiliations:** 1 School of Pharmaceutical Sciences, Guizhou University of Traditional Chinese Medicine, Dongqing Road, Huaxi District, Guiyang 550003, China; 18788705568@163.com (T.W.); 15338593427@163.com (C.L.); nc7901888@163.com (Y.N.); 2 College of Pharmacy, Huazhong University of Science and Technology, Wuhan 430000, China

**Keywords:** endophytic fungus, *Colletotrichum boninense*, secondary metabolites, *Polygonum multiflorum*

## Abstract

Endophytic fungi associated with medicinal plants constitute a promising source of structurally diverse and biologically active natural products. In this study, we investigated the secondary metabolites of *Colletotrichum boninense* (Strain H10), an endophyte isolated from the medicinal plant *Polygonum multiflorum* Thunb. A total of 25 secondary metabolites were isolated from rice-based fermentation cultures, among which three were new compounds: one ergosteroid (m1) and two solanapyrone-type polyketides (m2 and m3). The structures were elucidated via comprehensive spectroscopic analyses, including 1D/2D NMR, HRESIMS, ECD calculations, and X-ray crystallography. The known compounds comprised steroids, polyketides, alkaloids, and fatty acids. Phylogenetic analysis based on ITS and LSU sequences revealed that *Colletotrichum boninense* and related *Colletotrichum* strains form a distinct clade within the Glomerellaceae. These new solanapyrone and sterol derivatives support the chemotaxonomic significance of this class of compounds in *Colletotrichum* species. This work enriches the chemical diversity of endophytic fungi derived from *Polygonum multiflorum*.

## 1. Introduction

Endophytic fungi colonize healthy plant tissues, organs, and intercellular spaces, and persist across nearly the entire life cycle of host plants without causing any disease symptoms [1]. Endophytes play a vital role in producing bioactive natural products and can be considered as a strategically promising bio-resource with considerable economic potential for agrochemical and pharmaceutical industries [2,3]. Endophytic fungi are widely distributed in the plant kingdom, and more than 1.6 million endophytic fungal species have been reported from plant hosts [4,5]. Previous studies reveal that endophytic fungi often exert antimicrobial, antioxidant, and pest-resistance activities during plant growth and form synergistic symbiotic relationships with their hosts [6].

*Polygonum multiflorum* Thunb. (*P. multiflorum*) serves as the original plant of Polygoni Multiflori Radix, which is documented in Volume I of the Chinese Pharmacopoeia (2025 edition) [7]. Existing research on P. multiflorum Radix has largely concentrated on its cultivation, processing and preparation, chemical composition, and pharmacological activities. In comparison, studies concerning secondary metabolites from endophytic fungi inhabiting *P. multiflorum* are still relatively limited [8].

The diversity and abundance of endophytic fungi isolated from *P. multiflorum* remain limited, and only a small number of bioactive strains have been discovered, which warrants further exploration. Previous studies have shown that plants growing in warm and humid regions host richer endophytic fungal communities. Herein, *P. multiflorum* samples were collected from Guizhou Province, a warm and humid region in southwest China. The warm and humid climate of this region favors high endophytic fungi diversity associated with this medicinal plant [9,10].

Fungi of the genus *Colletotrichum* are destructive post-harvest pathogens infecting numerous plants, such as cereals, legumes, and fruits. Nevertheless, many *Colletotrichum* species (*Colletotrichum* sp.) can live as endophytes within asymptomatic plant tissues. A single strain is capable of switching between endophytic and pathogenic lifestyles according to host physiological status and environmental conditions [11,12]. The *Colletotrichum* sp. belong to a branch of the order Melanconiales in the subphylum Deuteromycotina. It has been reported that the metabolites produced by these fungi display various biological activities [13]. Up to now, at least 109 secondary metabolites have been reported from the fungal anthrax bacteria, including nitrogenous metabolites, sterols, terpenes, pyranones, phenols and fatty acids, mostly nitrogenous compounds [14]. Although the total count of terpenoids obtained from *Colletotrichum* remains relatively low, new discoveries are increasing rapidly and several monoterpenes, sesquiterpenes, and diterpenes have now been listed [13,15,16]. In a screening for novel anti-glycation agents, four new acorane-type sesquiterpenes were found, together with seven known compounds from *Colletotrichum gloeosporioides*, an endophyte derived from Sabicea cinerea. Their structures were elucidated by MS and nuclear magnetic resonance (NMR) spectroscopy, while their absolute configurations were established via circular dichroism (CD) analysis [17].

At present, most of the studies on *P. multiflorum* focus on its cultivation, medicinal materials processing, the composition and content of active ingredients in medicinal materials, and the related pharmacology of medicinal materials, while there are relatively few studies on endophytic fungi in *P. multiflorum*. The types and quantities of endophytic fungi isolated from *P. multiflorum* plants are limited, and the active strains screened out are also few, which requires further exploration. Previous studies have revealed that plants in warm and humid areas contained a greater abundance of endophytic fungi, and the *P. multiflorum* specimens in this study were collected from Guizhou Province [18]. Owing to Guizhou’s humid climatic conditions, the endophytic fungi inhabiting these plants are more abundant.

**Figure 1:**
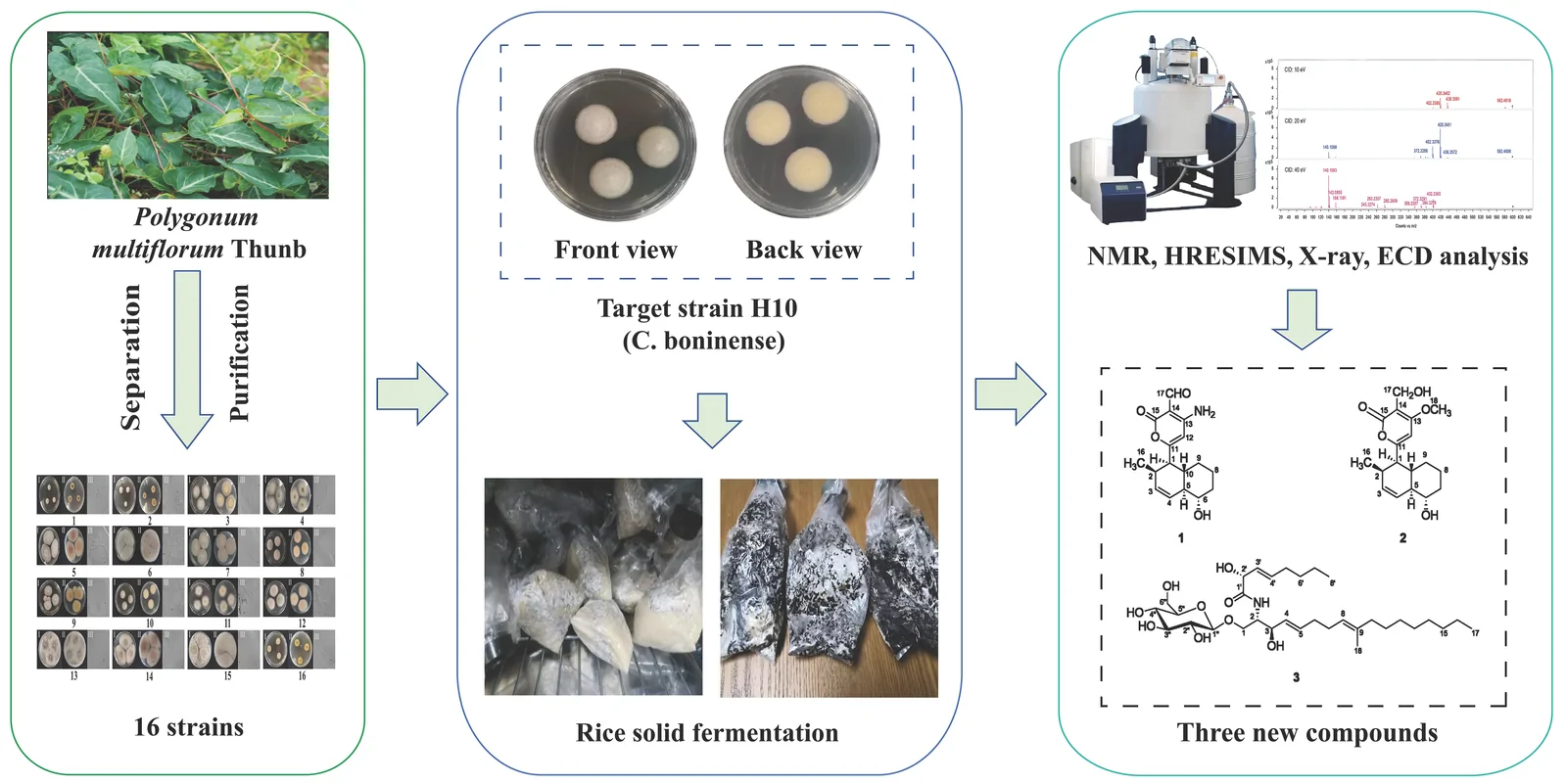
Research plan on secondary metabolites from fungi associated with *P. multiflorum*.

To explore the diversity and secondary metabolite resources of endophytic fungi associated with *P. multiflorum*, internal transcribed spacer (ITS) and large subunit (LSU) sequences were used to construct a dataset for phylogenetic analysis. Among the endophytic fungi isolates from *P. multiflorum*, strain H10 was identified, sharing 100% sequence similarity to *Colletotrichum boninense* (*C. boninense*). Given that this fungus has been rarely studied, strain H10 was chosen to investigate its secondary metabolites [19]. After fermentation, followed by metabolite isolation, purification and structural characterization, its secondary metabolites were dominated by steroids, fatty acids, polyketides, and alkaloids, among which three novel compounds were characterized. This work preliminarily clarified the chemical profiles of secondary metabolites from *P. multiflorum* endophyte, expanded natural medicinal resources, and provided a theoretical basis and practical guidance for further developing endophytic fungi from *P. multiflorum* as a new source of natural products (Figure 1).

## 2. Results

### 2.1. Phylogenetic Analysis

The lengths of the individual alignments used for the identification of endophytic fungi from *P. multiflorum* were 634 bp for ITS and 891 bp for LSU. The combined dataset of ITS and LSU sequences was used for phylogenetic analysis. The obtained sequences were submitted to the NCBI GenBank database and compared using BLAST (https://blast.ncbi.nlm.nih.gov/Blast.cgi) search against reference sequences. The nucleotide similarity threshold for species identification was set between 97% and 100% [20,21].

Among the 16 isolated endophytic fungal strains, three strains (H4, H10, H16) showed 100% sequence similarity with their respective reference strains, allowing identification at the species level. Strain H4 was identified as *Colletotrichum crassipes* (100% similarity), H10 as (*C. boninense*) (100%), H16 as *Colletotrichum destructivum* (100%). The remaining strains showed similarity values ranging from 99.0% to 99.98% with their closest matches, but due to the lack of uniqueness in the matches, they were identified only to the genus level (Table 1).

**Figure 2:**
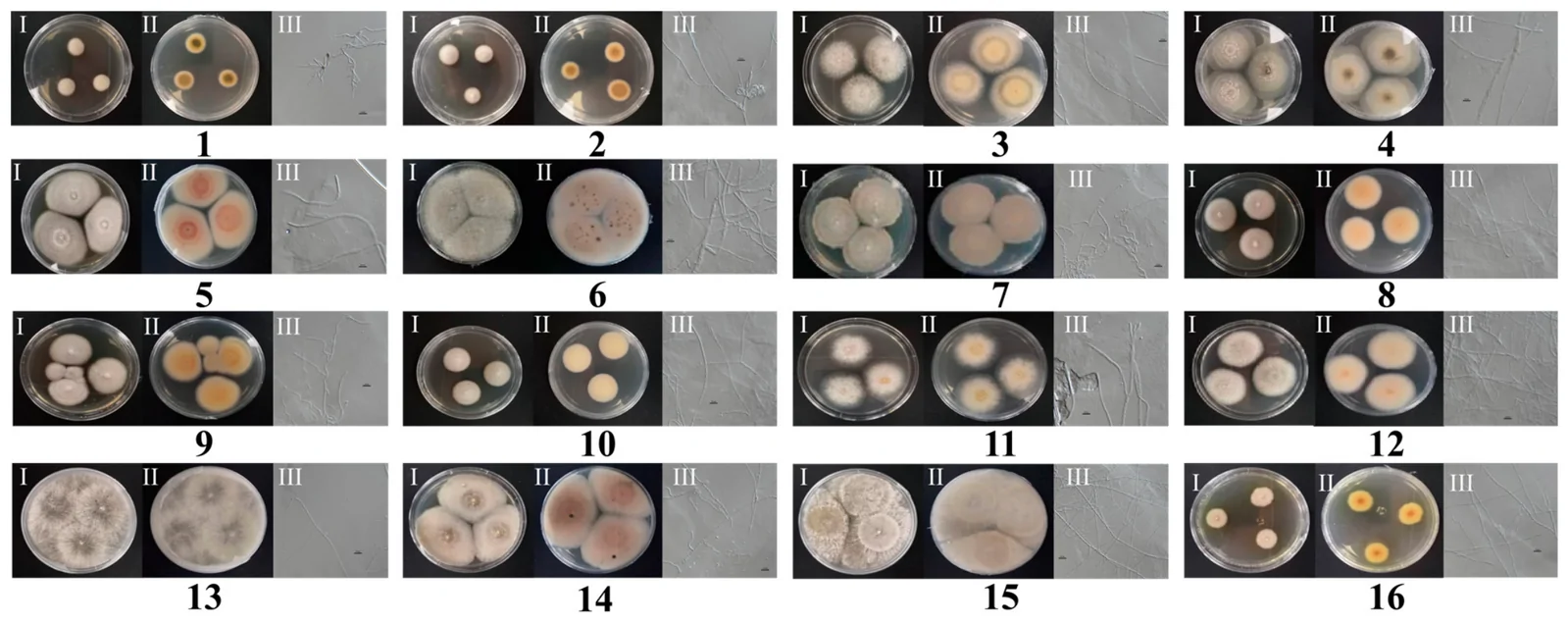
Results of isolation and purification of fungi from *P. multiflorum*. (Note: I, front view of colony; II, reverse view of colony; III, microscopic morphology of colony at 40/0.65× magnification.)

The phylogenetic analysis revealed that the isolated endophytic fungi belonged to five different families: *Pleosporineae*, *Glomerellaceae*, *Diaporthaceae*, *Ceratostomataceae*, and *Nectriaceae*. These families were further distributed across six genera: *Phaeosphaeria*, *Colletotrichum*, *Alternaria*, *Diaporthe*, *Harzia*, and *Dactylonectria*. Among these, *Colletotrichum* was the dominant genus, accounting for 62% (10 out of 16 strains) of all isolated endophytic fungi (Figure 2 and Table 1). Given the unique morphology and type of strain H10, a literature search revealed that there are currently few reports on the secondary metabolites of *C. boninense* strains isolated from *P. multiflorum*. Therefore, H10 (*C. boninense*) was ultimately selected for rice solid-state fermentation.

### 2.2. Taxonomy

Morphological description: Fungal colonies on potato dextrose agar (PDA) at 28 °C for 5–7 days. Colonies of H10: Colonies large, loose, cottony to floccose, dry, opaque, firmly attached to the medium, surface white to pale pink or grayish, reverse pale yellowish to brownish; mycelium septate, hyaline to pale brown, branched; growth rate fast, reaching 60–70 mm diameter after 7 days; odor moldy; no spores observed under light microscopy (40×).

Molecular identification: Genomic DNA was extracted from fresh mycelia, and the ITS region and LSU of the nuclear ribosomal RNA gene were amplified by polymerase chain reaction (PCR) using primer pairs ITS4/ITS5 and LR0R/LR5, respectively. The obtained sequences were submitted to NCBI GenBank and compared using BLAST search. Strain H10 showed 100% sequence similarity with *C. boninense* (GenBank accession number not assigned). Based on the combined morphological and molecular data, strain H10 was identified as *C. boninense*.

Habitat and distribution: Isolated from fresh plants of *P. multiflorum* collected from Wudang District, Guiyang, Guizhou Province, China (longitude 106.836223° E, latitude 26.791220° N). Strain H10 was isolated from the leaf.

Phylogenetic placement: The ITS and LSU sequences of the isolated strains were aligned with reference sequences from NCBI. The phylogenetic analysis showed that *Colletotrichum* species are the dominant endophytic fungi in *P. multiflorum*, accounting for 62% of all isolated strains (10 out of 16 strains). They belong to the family *Glomerellaceae*, order *Glomerellales*, class *Sordariomycetes*. The genus *Colletotrichum* is characterized by its fast-growing colonies, diverse pigmentation (white, pink, gray, or black) and the absence of visible spores under standard culture conditions, which is consistent with previous reports of endophytic *Colletotrichum* species.

### 2.3. Isolation of Compounds ***4***–***25***

**Figure 3:**
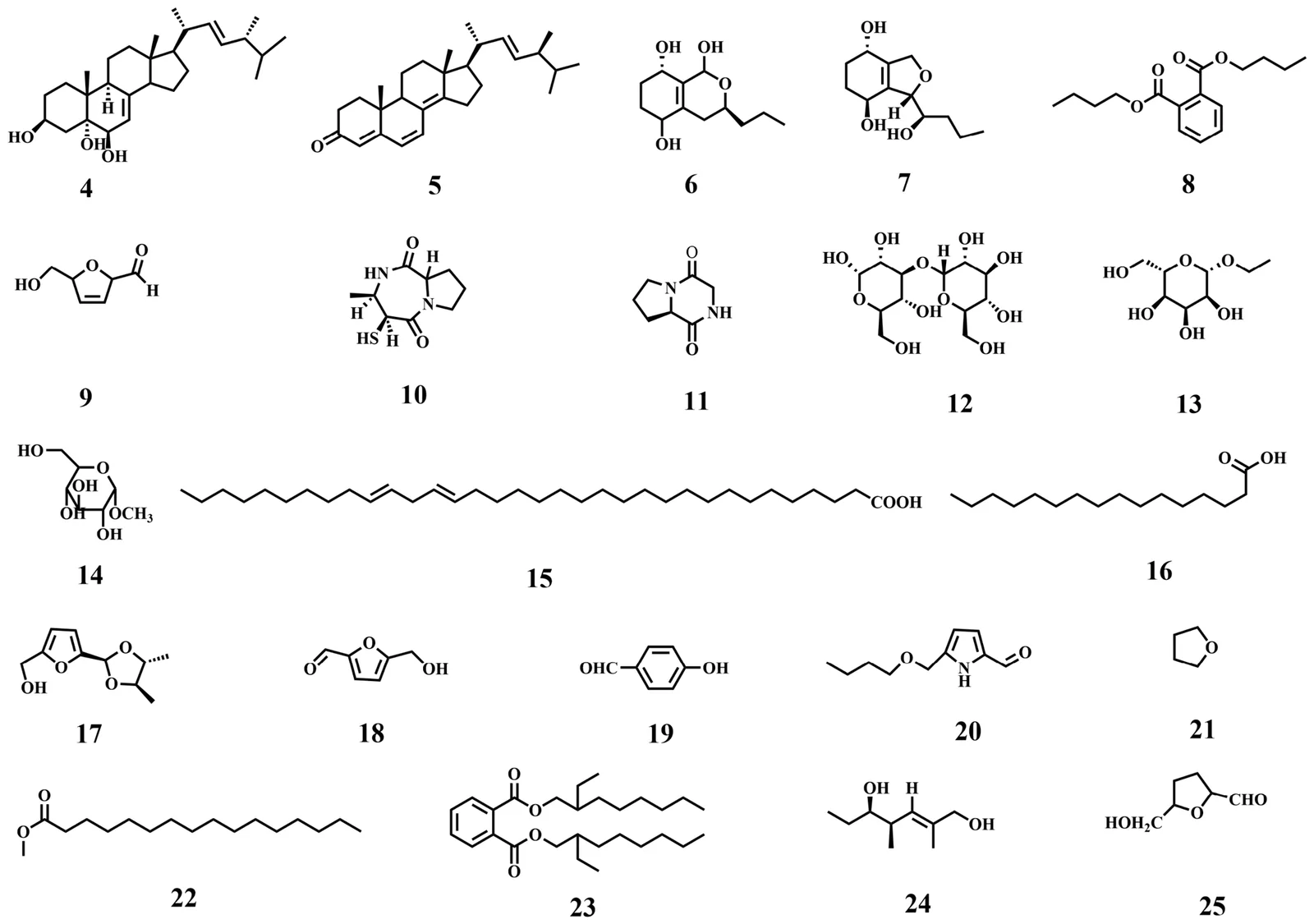
The chemical structures of compounds **4**–**25**.

The H10 strain was fermented, and its metabolites were isolated and purified using silica gel column chromatography, Sephadex LH-20 gel column chromatography and preparative HPLC. Based on HR-ESI-MS, ^1^H NMR, ^13^C NMR and DEPT spectral data as well as literature comparison, a total of 22 known compounds were obtained, including steroids, polyketides, phthalates, furan derivatives, nitrogen-containing compounds, saccharides, fatty acid/fatty alcohol derivatives and other small-molecule metabolites; their molecular formula, physical state and identification results are summarized in Table 2, and their structures are shown in Figure 3.

### 2.4. Identification of New Compounds

**Figure 4:**
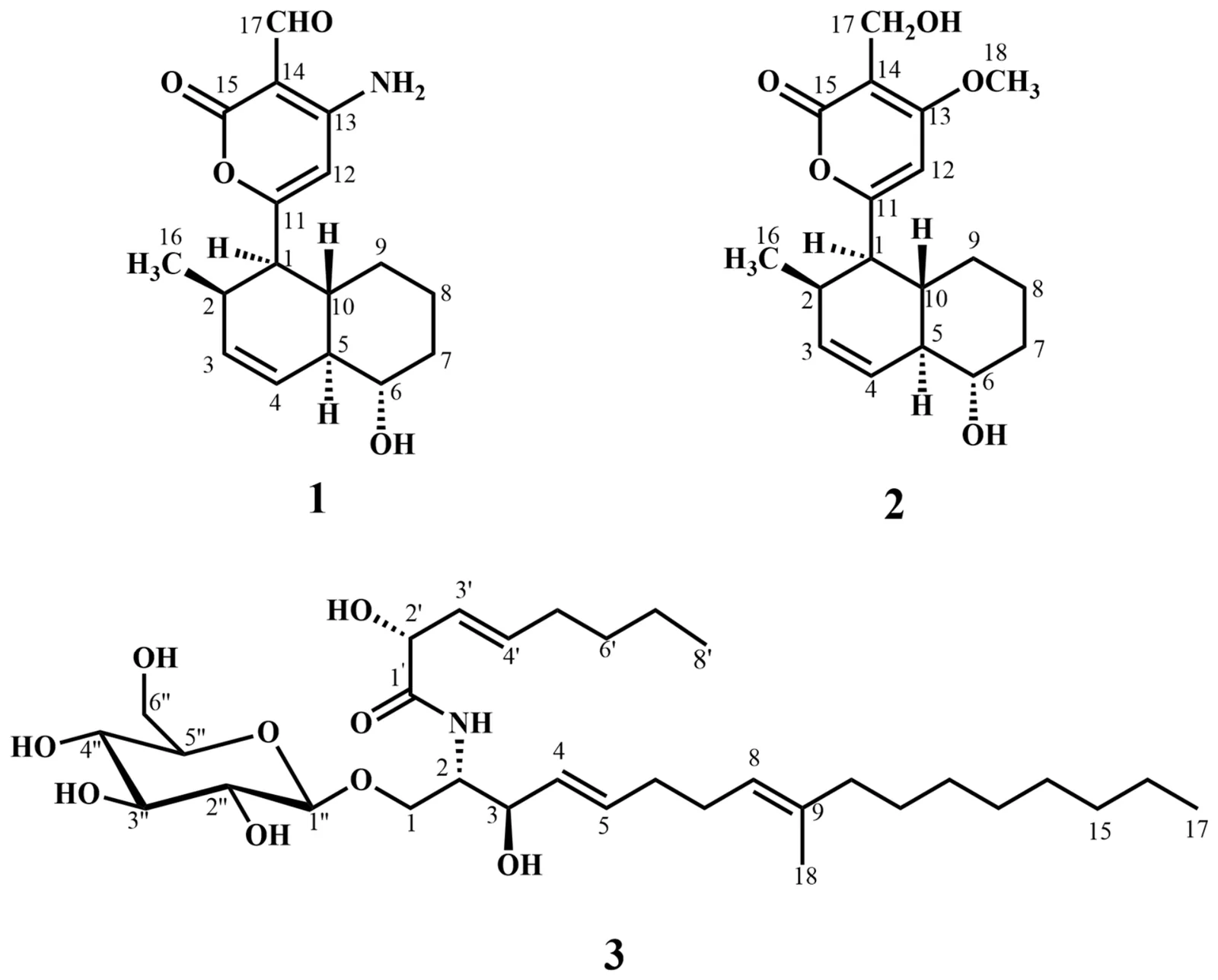
The chemical structures of compounds **1**–**3**.

Compounds **1**–**3** were obtained from the rice solid-state fermentation culture of strain H10, and their structures were identified by NMR and MS analyses as described in Section 4.4, “Fermentation and Extraction.” No information on compounds **1**–**3** was previously found in the literature or in the Dictionary of Natural Products (https://dnp.chemnetbase.com/, accessed on 15 September 2026), Reaxys database (https://www.reaxys.com/, accessed on 15 September 2026), PubChem database (https://pubchem.ncbi.nlm.nih.gov/, accessed on 15 September 2026) or CAS SciFinder Database (https://www.cas.org/, accessed on 15 September 2026). The chemical structures of compounds **1**–**3** are shown in Figure 4.

Compound **1** was isolated as a yellow oil. Its molecular formula C_17_H_21_NO_4_ was deduced from high-resolution electrospray ionization mass spectrometry (HRESI-MS) *m*/*z* 326.1361 [M+Na]^+^ [calculated (calcd) for C_17_H_21_NO_4_Na, 326.1368] and the molecular formula also suggests that the compound has eight unsaturated Indices of Hydrogen Deficiency (IHD). The infrared (IR) spectrum of 1 showed obvious absorption bands at 3416.27, 3319.08, 1522.96, and 1666 cm^−1^ and revealed the presence of corresponding hydroxyl, carbonyl and amino groups in compound **1**. The 1H nuclear NMR spectrum (Table 3) of 1 showed there is a methyl signal [δH 1.00, d (*J* = 7.2 Hz) H3-16] and three methylene signals [δH 2.05 and 1.30, m, H2-7, δH 1.79 and 1.44, m, H2-8, δH 1.68 and 0.91,m, H2-9]; in the low field region there are five proton hydrogen signals [δH 2.63, dd (*J* = 10.8, 6.0 Hz), H1-1, δH 5.67, ddd; (*J* = 10.2, 4.5, 2.2 Hz), H1-3, δH 5.99, br.d; (*J* = 9.8 Hz), H1-4, δH 3.32, td; (*J* = 9.5, 4.3 Hz), H1-6, δH 5.64, s, H1-12]. The compound has proton signals of five terminal olefins in the high field region [δH 2.63, m, dd (*J* = 10.8,6.0 Hz), H1-1 δH 2.46, m, H1-2, δH 1.73, m, H1-5, δH 3.32, td (*J* = 9.5, 4.3 Hz), H1-6, δH 1.70, H1-10]. 13C-DEPT NMR and HSQC spectra showed that the compound had 17 carbons, which were one methyl carbon, three methylene carbon signals, nine hypomethyl signals, and four quaternary carbon signals. The compound is consistent with the main data and is presumed to be a tricyclic phenolic acid compound [44].

**Figure 5:**
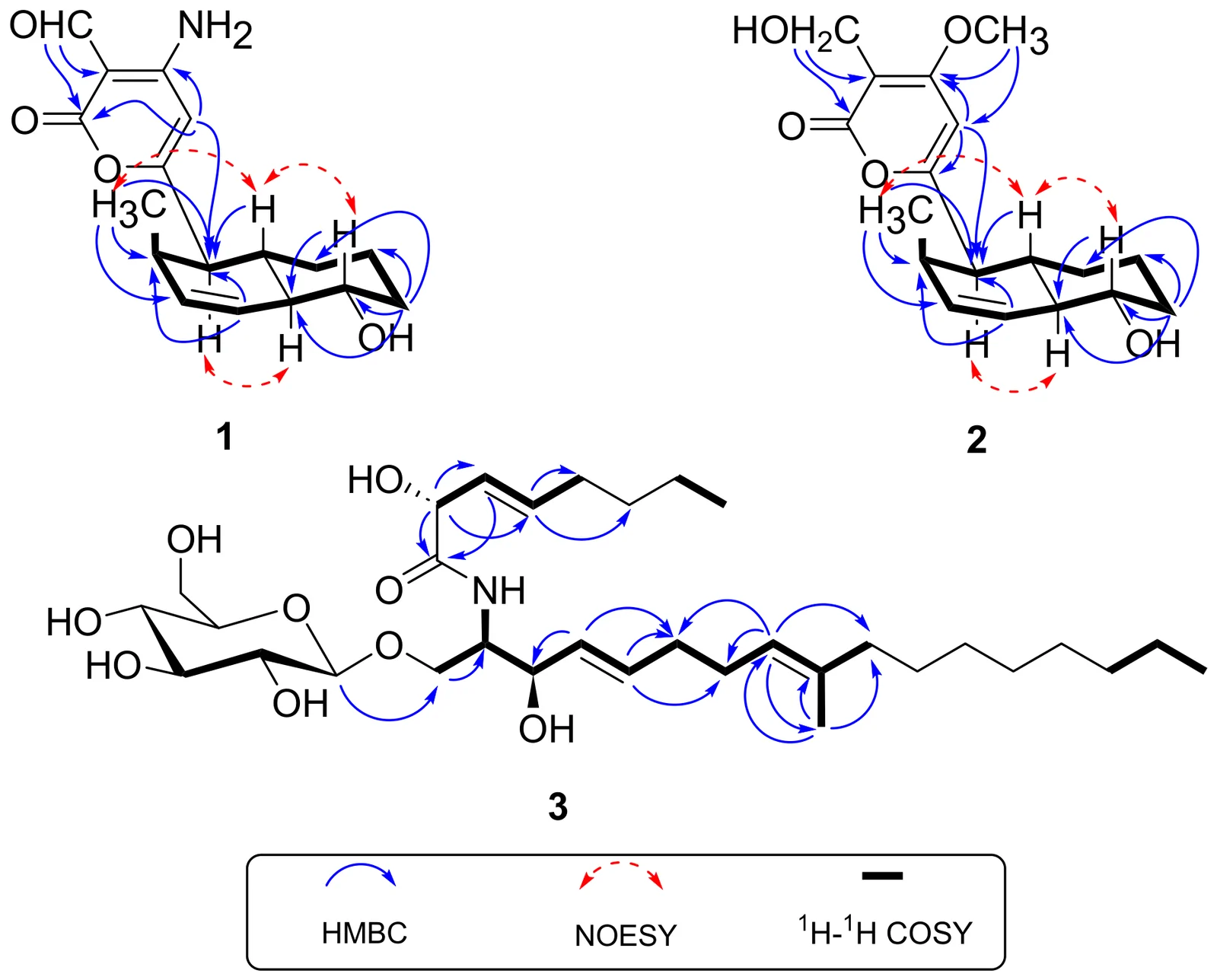
Key 1H-1H COSY, NOESY and HMBCs of compounds **1**–**3**.

**Figure 6:**
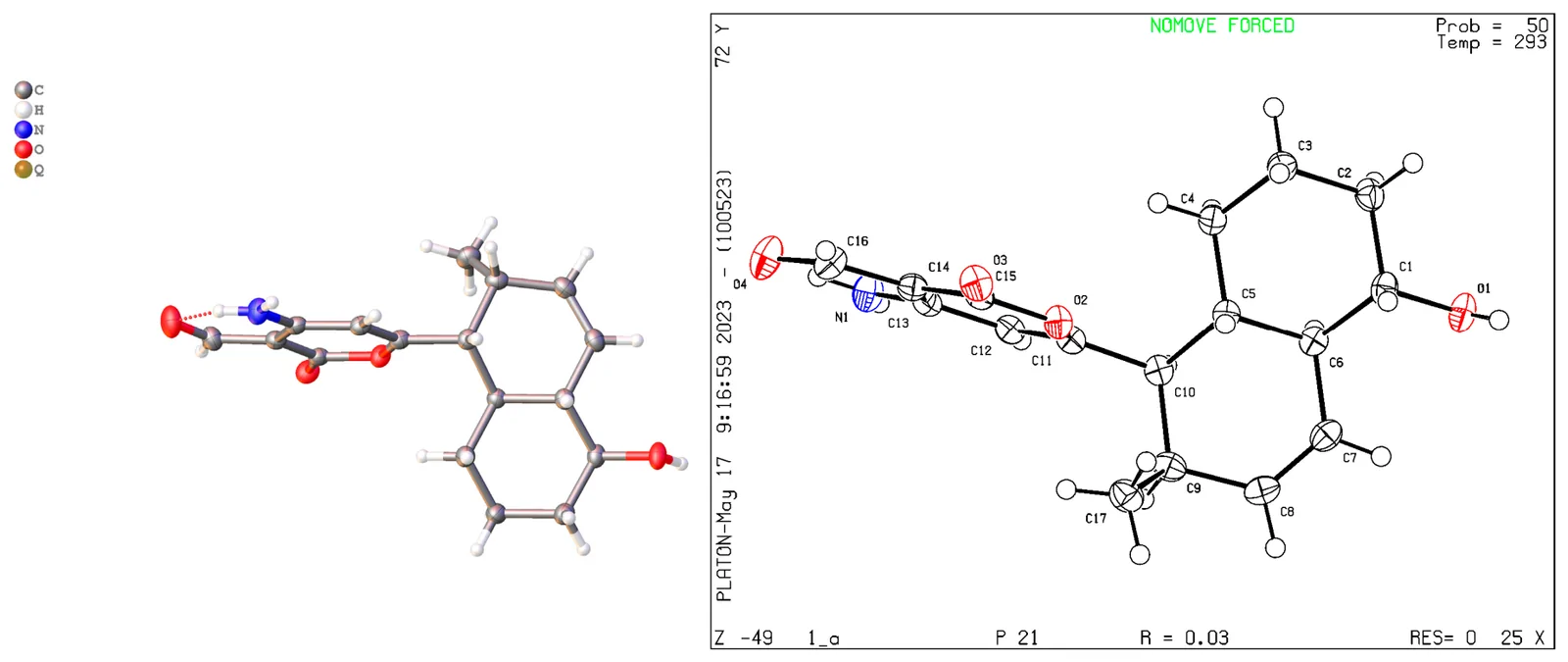
ORTEP drawing of the X-ray structure of Compound **1**.

Moreover, the significant heteronuclear multiple bond correlations (HMBCs) (Figure 5) from H3-16 to C-1 (δC 49.4), C-2 (δC 35.5), and C-3 (δC 132.3), H2-7 to C-5 (δC 49.9), C-6 (δC 73.2), C-8 (δC 24.1), and C-9 (δC 29.5), H-4 to C-1 (δC 49.4) and C-2 (δC 35.5), H-6 to C-5 (δC 49.9), H-10 to C-1 (δC 49.4), and H-17 to C-14 (160.1) and C-15 (δC 171.8) strongly suggested the existence of three penta-substituted benzene rings and the location of the aldehyde group of the compound is deduced. The relative configuration of 1 was determined by the nuclear Overhauser effect spectroscopy (NOESY) experiment Figure 5. As shown in Figure 5, the NOESY correlations of H-6 with H-10 and H3-16 revealed that these protons were on the same face and assumed as β-oriented, while the correlations of H-1 with H-5 indicated that they were α-oriented. In addition, on the basis of the 1H-1H correlation spectroscopy (COSY) fragment of C-1/C-17, the obvious HMBCs from H3-16 to C-1 (δC 49.4), C-2 (δC 35.5), and C-3 (δC 132.3), H2-7 to C-5 (δC 49.9), C-6 (δC 73.2), C-8 (δC 24.1), and C-9 (δC 29.5), H-4 to C-1 (δC 49.4) and C-2 (δC 35.5), H-9 to C-5 (δC 49.9), and H-10 to C-1 (δC 49.4), coupling with H-1 to C-11 (δC 163.7), conclusively confirmed the planar structure of m1 as shown in Figure 4. In order to further clarify the absolute configuration, electronic circular dichroism (ECD) calculation of m1 was performed on mPW1PW91/SVP level of theory. As a result, the experimental ECD spectrum perfectly matched with the calculated ECD spectrum configuration for m1. Therefore, the planar structure of 1 was then determined. The relative and absolute configurations of compound **1** were unambiguously confirmed by the X-ray single crystal diffraction on the CuKa with a Flack parameter of −0.05. Finally, the absolute configuration was thus designated as shown in Figure 6, and given the trivial name m1.

Compound **2** was isolated as a yellow oil, HRESI-MS: *m*/*z* 343.1518 [M+Na]^+^. The molecular formula is derived as C_18_H_24_O_5_ (calculated value of C_18_H_24_O_5_Na is 343.1512), and the molecular formula also suggests that the compound has seven unsaturated indices (IHD). IR (KBr) νmax 3386.43, 2918.10, and 1682.96 cm^−1^ presumed the presence of hydroxyl and carbonyl groups in compound **2**. As shown in Table 3, the 1H-NMR spectrum of the compound shows a methyl signal [δH 0.98, d (*J* = 7.2), H3-16], a methoxy signal [δH 3.91, s, H3-18], four methylene signals [δH 2.05, m, 1.31, m, H2-7, δH 1.78, m, 1.45, m, H2-8, δH 1.60, m, 0.88, m, H2-9, δH 3.91, s]; in the low field region there are four proton hydrogen signals [δH 2.74, dd (*J* = 11.1, 6.0 Hz), H1-1, δH 5.68, ddd (*J* = 10.2, 4.5, 2.0 Hz), H1-3, δH 6.00, br.d (*J* = 10.0 Hz), δH 6.08, s]. The compound has proton signals of five terminal olefins in the high field region [δH 2.46, m, H1-2, δH 1.75, m, H1-5, δH 1.73, m, H1-10]. ^13^C-DEPT NMR spectra showed that the compound had 18 carbons, which were two methyl carbon, four methylene carbon signals, eight hypomethyl signals, and four quaternary carbon signals (one aldehyde carbon signal and one carbonyl signal). The compound is consistent with the main data and is presumed to be a tricyclic phenolic acid compound [45].

**Figure 7:**
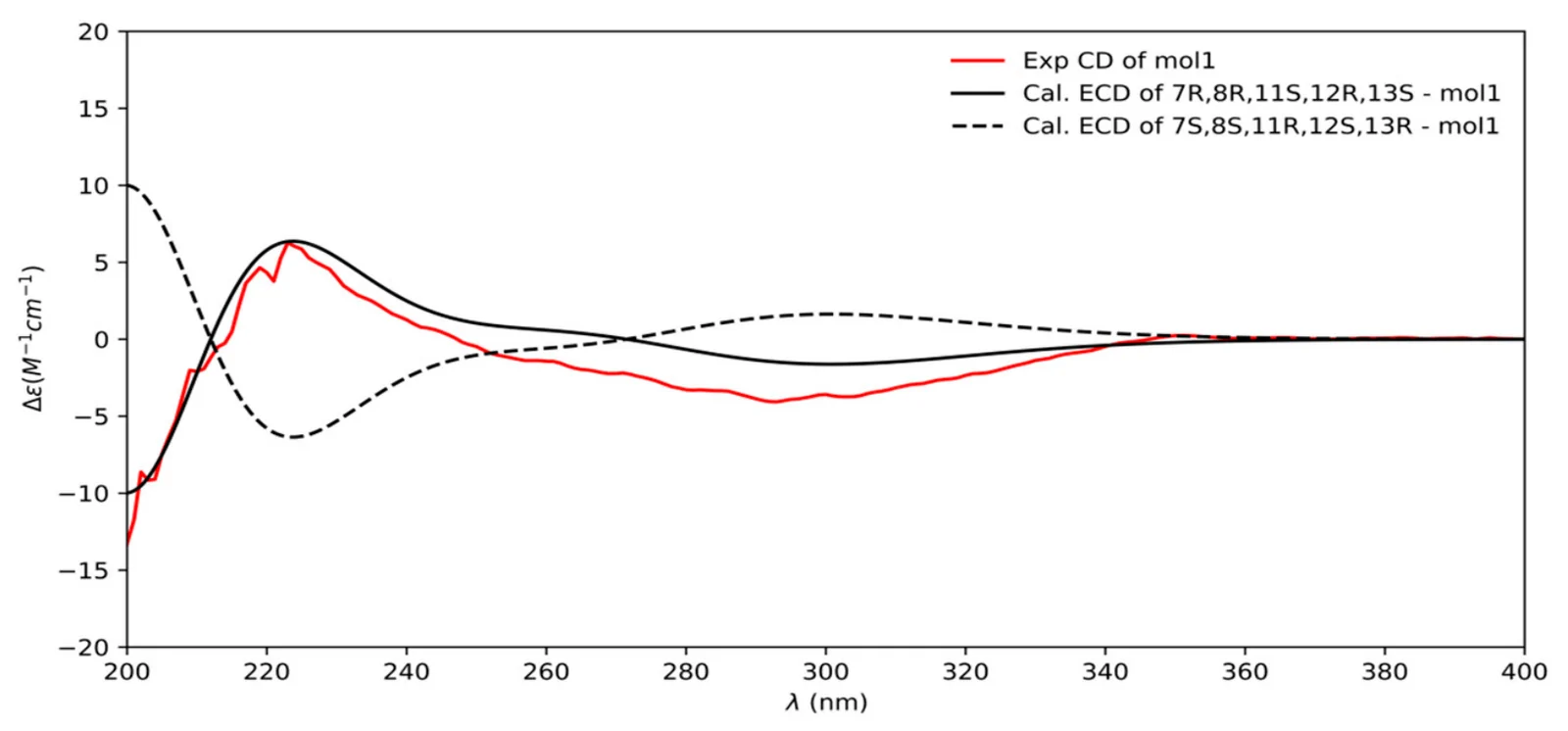
Experimental ECD spectra (200-400 nm) of mol 1 in methanol and the calculated ECD spectra of the model molecules of mol 1 at the PBE0/def2-TZVP level.

The planar structure of the compound can be inferred from two-dimensional NMR data, and its HMBC spectrum shows that H-3 is associated with methyl (Figure 5), C-1, and C-5, H-5 is associated with C-1, C-7, and C-9, H-7 is associated with C-5, C-9, and C-10, H-9 is associated with C-1, C-7, and C-11, H-1 is associated with methyl, C-5 and C-12, H-12 is associated with C-1, C-11, C-14, and O-Me, and H-CH2OH is associated with C-14. The C-15 correlation determines the location of the carbonyl group and O-Me. According to HMBC, the compound is a ternary ring, and according to 1H-1H COSY H-5 is associated with H-10, H-4, and H-6, H-10 is related to H-5, H-1, and H-9, H-1 is associated with H-11, H-10, and H-2 phases, H-3 is related to H-2 and H-4, H-2 is related to methyl, the C ring is connected by C-1 and C-11, and the location of the methyl group is introduced according to 13C-DEPT NMR spectra, which showed that C-6 was a hypomethyl carbon signal and C-6 was linked to a hydroxyl group according to the chemical shift value, thereby determining the ligation order of the three six-membered rings. According to the NOESY signal, H-5 is related to H-1, H-6, and H-10, H-1 is related to H-2, H-6 is related to H-7, H-5, and H-1, and finally the configuration is determined by analyzing the data of compound **2** and the measured ECD data as shown in Figure 7, and it was revealed as a new, natural, rarely occurring compound, which was given the trivial name m2.

**Figure 8:**
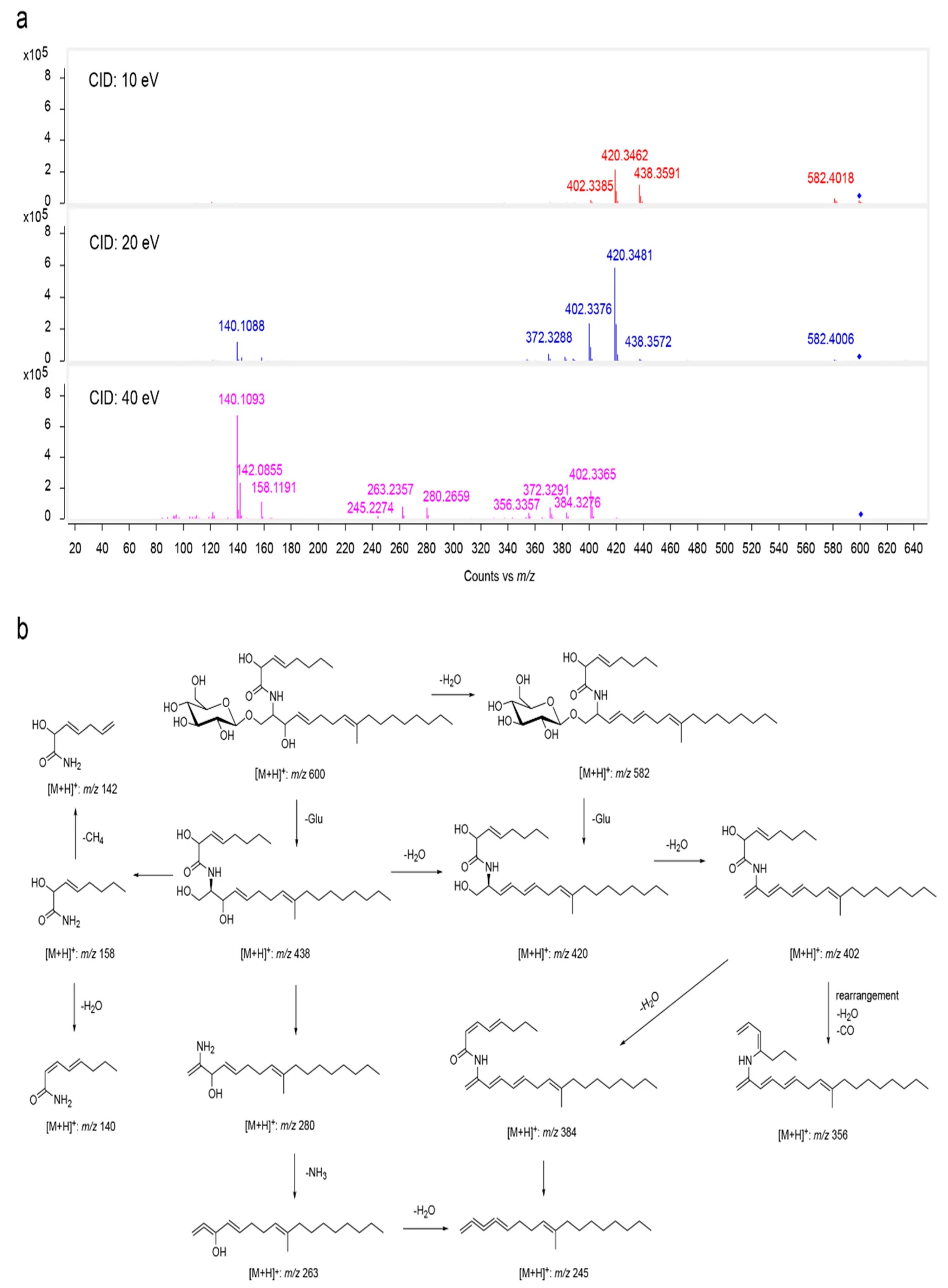
MS/MS analysis of compound **3**. (**a**) Detailed ESI-MS/MS in positive ion mode with the CID of 10, 20 and 40 eV, respectively. (**b**) The proposed fragmentation pathway of compound **3**.

Compound **3** was isolated as a white powder. IR (KBr) νmax 3398, 2922, 1653, and 1073 cm^−1^ presumed the presence of hydroxyl, carbonyl and amino groups in compound **3**. Its molecular formula C_32_H_57_NO_9_ was deduced from HRESI-MS: *m*/*z* 622.3904 [M+Na]^+^ [calculated (calcd) for C_32_H_57_NO_9_Na, 622.3931], and the molecular formula also suggests that the compound has five unsaturated indices of hydrogen deficiency (IHDs) and MS/MS analysis of compound **3**. (a) Detailed ESI-MS/MS in positive ion mode with the CID of 10, 20 and 40 eV, respectively. (b) The proposed fragmentation pathway of compound **3** (Figure 8). As shown in Table 4, the 1H-NMR spectrum of the compound shows three methyl signals [δH 0.85, d (*J* = 7.0), H3-17, δH 1.54, s, H3-18, δH 0.85, t (7.0), H3-8′] and 15 methylene signals [δH 4.05, m, H2-1, δH 1.96, m, H2-6, δH 1.97, m, H2-7, δH 5.06, br. s, H2-8, δH 1.91, t (6.0), H2-10, δH 1.22-1.31, H2-(11-14), δH 1.22, m, H2-15, δH 1.24, m, H2-16, δH 1.96, m, H2-5′, δH 1.22, m, H2-6′, δH 1.28,m, H2-7′, δH 3.83, d (*J* = 11.2 Hz), H2-6′’]. In the low field region there are six proton hydrogen signals [δH 5.38, dd (*J* = 15.0, 7.2 Hz), H1-4, δH 5.68, dd (*J* = 15.0, 5.6 Hz), H1-5, δH 4.49, d (*J* = 5.4 Hz), H1-2′, δH 5.40, dd (*J* = 15.0, 5.8 Hz), H1-3′, δH 5.79, dt (*J* = 14.8, 7.0 Hz), H1-4′, δH 4.33, d (*J* = 7.7 Hz), H1-1″]. The compound has proton signals of six terminal olefins in the high field region [δH 3.95, m, H1-2, δH 3.37, m, H1-3, δH 3.25, m, H1-1″, δH 3.44, ma, H1-3″, δH 3.30, ma, H1-4″, δH 3.36, ma, H1-5″]. 13C-DEPT NMR spectra showed that the compound had 32 carbons, which were three methyl carbon, 15 methylene carbon signals, eight hypomethyl signals, two quaternary carbon signals, and one amino signal. The compound is consistent with the main data and is presumed to be a enamide compound [46,47,48].

Detailed 2D-NMR studies (HSQC, 1H-1H-COSY, and HMBC experiments) afforded the gross structures as depicted in Figure 5 1H-1H-COSY correlation between H-7′, H-15 and H-8′, H-18, and HMBCs from H3-18 are related to C-10, C-9, and C-8, from H-8 is related to C-6, C-7, C-10, and C-18, and H-2′ is related to C-1′, C-3′, and C-4′, from H-3′ is related to C1′, and from H-4′ is related to C-5′, and C-6′; further, there are three methyl groups, three double bonds, and one carbonyl group on the ring, and the coupling of H-1 to C-10 conclusively confirmed the planar structure of m3 as shown in Figure 4. Thus, compound **3** was determined as depicted and given the trivial name m3. Finally, the configuration was determined by analyzing the data of compound **3** and the measured ECD data ergosterane.

m1: Yellow oil; [α]20 D +205.8° (c 0.001, MeOH); UV (MeOH) λmax (logε) 232 (+3.8), 309 (+3.9); 1H-NMR and 13C-NMR see Table 3; H RESIMS (positive) *m*/*z* 326.1361 [M+Na]^+^ (calcd for C_17_H_21_NO_4_Na^+^, 326.1368).

m2: Yellow oil; [α]20 D -213° (c 0.001, MeOH); UV (MeOH) λmax (logε) 207 (4.6), 303 (3.6), 345 (3.8); 1H-NMR and 13C-NMR see Table 3; HRESIMS (positive) *m*/*z* 343.1518 [M+Na]^+^ (calcd for C_18_H_24_O_5_Na^+^, 343.1512).

m3: White, powder; [α] 20 D +514° (c 0.001, MeOH); UV (MeOH) λmax (logε) 281 (3.7); 1H-NMR and 13C-NMR see Table 4; HRESIMS (positive) *m*/*z* 622.3904 [M+Na]^+^ (calcd for C_32_H_57_NO_9_Na^+^, 622.3931).

## 3. Discussion

In this work, 16 strains of endophytic fungi were isolated from the medicinal plant *P. multiflorum* collected in Guizhou. Taxonomic identification revealed that *Colletotrichum* dominated the endophytic fungal community of this host, accounting for 62% of the total isolates. *Colletotrichum* is classified under phylum *Ascomycota*, order *Glomerellales*, and represents one of the most prevalent endophytic fungal groups associated with leaves and branches of tropical, subtropical, and warm-temperate forest plants. Warm and humid environments promote its spore germination, appressorium formation, and host tissue colonization [49,50]. Guizhou Province features a subtropical humid monsoon climate that supports the growth of *Colletotrichum* sp. Many reports have demonstrated that *Colletotrichum* frequently shows the highest isolation frequency from healthy host tissues, and the fungus is capable of lifestyles switching between endophytism and pathogenicity depending on environmental factors and host conditions [11]. Our observation of *Colletotrichum* from *P. multiflorum* samples collected in the humid climate zone of the Guizhou Plateau further supports the strong adaptability of this genus to warm and humid habitats and medicinal plant hosts. However, research on the secondary metabolites of *C. boninense* as an endophytic fungus remains limited; existing studies have mostly focused on its pathogenicity as a plant pathogenic fungus rather than its chemical diversity as an endophyte [13]. Accordingly, strain H10 was chosen as the target isolate, and a rice-based solid fermentation system was applied to explore its secondary metabolites, so as to aim to elucidate the chemical profile of this strain.

Twenty-five secondary metabolites were isolated and identified from strain H10, including three new natural products (m1–m3) and 22 known compounds; their structural types include steroids, polyketides, alkaloids, carbohydrates, and fatty acids. m1 is an ergostane-type derivative, whereas m2 and m3 are solanapyrone-type polyketides. Solanapyrone-type polyketides have long been reported mainly from phytopathogenic fungi such as Alternaria and Ascochyta. Their biosynthesis is governed by a canonical iterative type I polyketide synthase (iPKS) gene cluster (the sol gene cluster), with the terminal step catalyzed by the Diels–Alderase Sol5 to construct the bicyclic scaffold [50,51]. In recent years, such compounds have also been discovered in endophytic fungi associated with medicinal plants. For example, three new pyranone compounds, solanapyrones P–R, along with known solanapyrones and benzopyranones, were isolated from extracts of the endophytic fungus *Alternaria tenuissima* SP-07 from *Salvia miltiorrhiza* [52]. In addition, pyranone constituents such as solanapyrone G have been detected in the metabolite profile of *Alternaria tenuissima* [53]. In this study, compounds with this new scaffold were obtained from an endophytic *Colletotrichum* strain, not only expanding the range of fungal sources of solanapyrone-type metabolites but also providing new chemical evidence for the chemotaxonomy of the genus *C. boninense*, suggesting that these polyketides may serve as reference metabolites for species-level chemotaxonomy.

Endophytic fungi form symbiotic associations with their host *P. multiflorum*. Nevertheless, whether the steroidal and polyketide metabolites biosynthesized by strain H10 endow the host with stress tolerance or pest/disease resistance remains to be elucidated. In the preliminary investigation, only basic activity screening was conducted, and no remarkable bioactivity was observed. Earlier reports have shown that solanapyrone-type compounds exert phytotoxic, antibacterial, and antitumor activities [54], whereas ergostane-type derivatives often exhibit anti-inflammatory, immunomodulatory, and other activities [55]. Hence, future research should adopt broader screening models and carry out systematic biological activity evaluation of the three new compounds to uncover their biological roles and prospects for drug discovery.

This result has several limitations. First, only one culture regime—rice-based solid-state fermentation—was used. Fungal secondary metabolite biosynthesis is highly culture-dependent; variations in medium formulation, temperature, pH, or incubation duration may activate silent biosynthetic pathways and yield extra structurally unique metabolites. Second, only strain H10 was investigated. The other 15 endophytic fungal strains isolated from *P. multiflorum* have not yet been studied, and their chemical potential remains unexplored. Third, metabolite analysis was performed only under in vitro fermentation. It remains to be validated by metabolomics whether strain H10 produces these metabolites inside *P. multiflorum* plants under native in situ conditions and what their actual physiological concentrations are within the host.

In summary, this work expands the chemical resources of endophytic fungi from *P. multiflorum* and provides strain resources for the subsequent mining of metabolites from *Colletotrichum* endophytic fungi. Future work may apply the OSMAC (one strain many compounds) strategy to alter culture conditions to activate silent biosynthetic gene clusters. Simultaneously, genome mining can be combined to analyze the biosynthetic gene clusters of this strain and elucidate the biosynthetic pathways of the three new compounds. These efforts will lay a theoretical framework for the discovery and exploitation of novel natural products.

## 4. Materials and Methods

### 4.1. Laboratory Apparatus

NMR instrument: Bruker AM-600 NMR spectrometer (Bruker, Karlsruhe, Germany); Infrared spectrometer: Bruker Vertex 70 FT-IR spectrophotometer (Bruker, Karlsruhe, Germany); UV spectrometer: Varian Cary 50 UV/VIS spectrophotometer (Varian, Salt Lake City, UT, USA); Circular dichroism spectrometer: JASCO-810 spectrometer; High Resolution Mass Spectrometer: Thermo Fisher LTQ XL LC/MS (Thermo Fisher, Palo Alto, CA, USA); Single crystal diffractometer(Rigaku Corporation, Tokyo, Japan): Rigaku Oxford diffractometer equipped with graphite-monochromatized Cu Kα radiation source; Semi-preparation uses HPLC (Agilent Technologies, Santa Clara, CA, USA)(Agilent 1200) flow rate 2 mL/min.

### 4.2. Strain of the Material

Samples of *P*. *multiflorum* used in this study were collected near Chichonggou, Wudang District, Guiyang, Guizhou Province, China, and identified by Lisha Dong at Guizhou University of Traditional Chinese Medicine as Polygoni Multiflori Radix. *C. boninense* was subsequently isolated and purified from fresh *P*. *multiflorum* leaves.

### 4.3. Isolation and Identification of Endophytic Fungi

In this study, endophytic fungi were isolated and purified from the fibrous roots, tuberous roots, stems, and leaves of *P. multiflorum*. The materials were rinsed with sterile water and surface-sterilized. Root tissues were then cut into small pieces of approximately 0.5 cm^3^ and leaves into slices of approximately 0.5 cm^2^ using a sterile scalpel. The tissues were inoculated onto PDA solid medium and incubated at a constant temperature of 28 °C for 3–7 days. After hyphae grew at the contact site between the cut edge and the medium, hyphae were picked and purified by streaking to obtain endophytic fungi with distinct morphological characteristics. Before sampling, the scalpel was sterilized under ultraviolet (UV) light for 30 min. Subsequently, mycelia from purified strains were scraped in a sterile area beside an alcohol lamp flame and stored at −20 °C. The frozen mycelia were ground into a slurry for subsequent genomic DNA extraction. Genomic DNA was extracted according to the instructions of the EZgene Fungal Genomic DNA Extraction Kit (B518259)(Sangon Biotech, Shanghai, China), using morphologically intact fungal cultures. Molecular identification was performed by PCR(P&Q Science & Technology, Shanghai, Beijing, China) amplification of the fungal internal transcribed spacer (ITS) region and the ribosomal LSU rRNA gene. The ITS region was amplified using the primer pair ITS5/ITS4, and the LSU gene was amplified using the primer pair LR0R/LR5.

### 4.4. Fermentation and Extraction

The endophytic fungi strain H10 was initially grown on PDA at 28 °C for purification. For seed culture preparation, fungal plugs (1 × 1 cm) were inoculated into 250 mL Erlenmeyer flasks containing 100 mL of potato dextrose broth (PDB) and incubated at 28 °C on a rotary shaker at 140 rpm. After 5 days of cultivation, the seed culture was used for solid-state fermentation. For each strain, 30 kg of brown rice was used as the solid fermentation substrate. The rice was distributed into sterilized bags (50 g per bag), mixed with an equal volume of distilled water (1:1, *w*/*v*), and autoclaved at 121 °C for 30 min. After cooling to room temperature, each bag was inoculated with 6 mL of the seed culture under sterile conditions. The fermentation was carried out at 28 °C for 20 days.

For compound extraction, the fermented rice cultures were soaked in 70% ethanol for 30 days. The supernatant was collected and the mycelia and residual rice were further extracted once more with 70% ethanol. The combined ethanol extracts were concentrated in vacuo at 45 °C. The resulting aqueous residue was sequentially extracted with petroleum ether, ethyl acetate (EtOAc), and n-butanol (nBuOH). Each organic phase was separated and concentrated under reduced pressure to yield the respective crude extracts. For H10, the EtOAc extract was obtained. All crude extracts were stored at 4 °C prior to chromatographic separation.

### 4.5. Isolation of Secondary Metabolites of Fungi

The ethyl acetate and n-butanol fractions of the secondary metabolites from strain H10 were systematically isolated. The ethyl acetate fraction was first fractionated by 100–200 mesh normal-phase silica gel column(Qingdao Haiyang Chemical Co., Ltd., Qingdao, China) chromatography, with sequential gradient elution using two solvent systems: petroleum ether–ethyl acetate and dichloromethane–methanol. Similar fractions were combined to yield eight fractions. HPLC and TLC analyses showed that the compounds were mainly enriched in the dichloromethane:methanol 5:1 and petroleum ether:ethyl acetate 1:1 fractions. The dichloromethane:methanol 5:1 fraction was separated on an ODS column and repeatedly purified by semi-preparative HPLC to give the corresponding individual compounds. The dried extract of the n-butanol fraction was mixed with 100–200 mesh normal-phase silica gel at a ratio of 1:1.2 (*w*/*w*), loaded onto a 100–200 mesh normal-phase silica gel column, and subjected to gradient elution with a dichloromethane–methanol system (20:1, 10:1, 5:1, 3:1, 2:1, 1:1, and 1:2). The column was finally washed sequentially with ethyl acetate and methanol, yielding a total of nine fractions. Each fraction was analyzed by HPLC and TLC. The dichloromethane:methanol 5:1, 3:1, 2:1, and 1:1 fractions showed no obvious main peaks in HPLC or obvious main spots in TLC; therefore, the compound-enriched dichloromethane:methanol 20:1 (Fr20) and 10:1 (Fr10) fractions were selected for further separation. Fr10 was further subdivided by normal-phase silica gel column chromatography: Fr9 was recrystallized to give compound m1; Fr12 was purified by semi-preparative HPLC (60% acetonitrile–water, 15 min) to give compound m2. After Fr20 was subdivided by normal-phase silica gel column chromatography, its Fr9 was further separated on an ODS column(5 μm, 10 × 250 mm, YMC Co., Ltd., Kyoto, Japan) to afford 10 subfractions. Among these, Fr3 was purified by preparative HPLC (55% acetonitrile–water, 35 min) to give compound m3. The remaining impure fractions were selectively further purified, according to their composition, by Sephadex LH-20 gel column(GE Healthcare Bio-Sciences AB, Uppsala, Sweden) chromatography and repeated semi-preparative HPLC, ultimately affording the aforementioned individual compounds.

### 4.6. Identification of Fungal Secondary Metabolites

For each individual compound obtained after isolation, UV spectra (MeOH) were first measured to assess the conjugated system; infrared (IR) spectra (KBr) were recorded to identify functional groups such as hydroxyl, carbonyl, and amino groups; and specific rotations ([α]D) were determined. Subsequently, high-resolution electrospray ionization mass spectrometry (HR-ESI-MS) was used to deduce the molecular formula and degrees of unsaturation from the exact masses of [M+Na]^+^ or [M+H]^+^. One-dimensional nuclear magnetic resonance (1D NMR), including ^1^H NMR (600 MHz), ^13^C NMR (150 MHz), and DEPT, was then applied to assign carbon and proton signals, distinguish among methyl, methylene, methine, and quaternary carbons, and identify structural fragments such as carbonyls and double bonds. Two-dimensional NMR (2D NMR), including ^1^H–^1^H COSY, HMBC, and NOESY, was further employed to deduce the connectivity of adjacent fragments, the planar structure, and the relative configuration. For new compounds, ECD was additionally measured and compared with the calculated or literature ECD data to determine the absolute configuration. For compounds that could be grown into single crystals, single-crystal X-ray diffraction was used as a supplement to directly confirm their stereostructures. For known compounds, the obtained MS and NMR data were compared with the published literature data to directly determine their structures.

## Figures and Tables

**Table 1:** Identification of endophytic fungi isolated from *P. multiflorum*.

Strain No.	Family	Genus	Nearest Type Strain	Identity (%)
H1	*Pleosporineae*	*Phaeosphaeria*	*Phaeosphaeria* sp.	99.55
H2	*Pleosporineae*	*Phaeosphaeria*	*Phaeosphaeria herpotrichoides* sp.	99.66
H3	*Glomerellaceae*	*Colletotrichum*	*Colletotrichum* sp.	99.00
H4	*Glomerellaceae*	*Colletotrichum*	*Colletotrichum crassipes*	100.00
H5	*Glomerellaceae*	*Colletotrichum*	*Colletotrihum fioriniae* sp.	99.98
H6	*Glomerellaceae*	*Colletotrichum*	*Colletotrichum gloeosporioides f.* sp.	99.54
H7	*Glomerellaceae*	*Colletotrichum*	*Colletotrichum fioriniae* sp.	99.76
H8	*Glomerellaceae*	*Colletotrichum*	*Colletotrichum johnstonii* sp.	99.88
H9	*Glomerellaceae*	*Colletotrichum*	*Colletotrichum costaricense* sp.	99.88
H10	*Glomerellaceae*	*Colletotrichum*	*C. boninense*	100.00
H11	*Glomerellaceae*	*Colletotrichum*	*Colletotrichum* sp.	99.43
H12	*Pleosporineae*	*Alternaria*	*Alternaria alternata* sp.	99.89
H13	*Diaporthaceae*	*Diaporthe*	*Diaporthe helianthi* sp.	99.44
H14	*Ceratostomataceae*	*Harzia*	*Harzia velata* sp.	99.53
H15	*Nectriaceae*	*Dactylonectria*	*Dactylonectria macrodidym* sp.	99.88
H16	*Glomerellaceae*	*Colletotrichum*	*Colletotrichum destructivum*	100.00

**Table 2:** Information of the identified compounds.

No.	Identified Name	Molecular Formula	Appearance	Compound Class	Reference
**4**	cerevisterol	C_28_H_46_O_3_	yellow oil	steroidal compound	[22]
**5**	10,13-Dimethyl-17-(1,4,5-trimethyl-hex-2-enyl)-1,2,9,10,11,12,13,15,16,17-decahydrocyclopenta[a]phenanthren-3-one	C_28_H_40_O	yellow oil	steroidal compound	[23]
**6**	letendronol A	C_12_H_20_O_4_	yellow oil	polyketide	[24]
**7**	letendronol D	C_12_H_20_O_4_	yellow oil	polyketide	[25]
**8**	di-n-butyl phthalate	C_16_H_22_O_4_	colorless oil	phthalate	[26]
**9**	Hex-3-enose, 2,5-anhydro-3,4-dideoxy- (9CI)	C_6_H_8_O_3_	yellow oil	furan aldehyde	[27]
**10**	3-methyl-4-sulfanyl-3,4,7,8,9,9a-hexahydro-2H-pyrrolo[1,2-a][1,4]diazepine-1,5-dione	C_9_H_14_N_2_O_2_S	yellow oil	nitrogen-containing compound	[28]
**11**	(R)-hexahydropyrrolo[1,2-a]pyrazine-1,4-dione	C_7_H_10_N_2_O_2_	yellow oil	nitrogen-containing compound	[29]
**12**	α-nigerose	C_12_H_22_O_11_	white crystalline powder	sugar	[30]
**13**	α-ethyl glucoside	C_8_H_16_O_6_	white powder	sugar	[31]
**14**	methyl-α-D-glucopyranoside	C_7_H_14_O_6_	white powder	sugar	[32]
**15**	n-tetratriacont-20,23-dienoic acid	C_34_H_64_O_2_	yellow oil	fatty acid and fatty alcohol derivative	[33]
**16**	palmitic acid	C_16_H_32_O_2_	white plate crystals	fatty acid and fatty alcohol derivative	[34]
**17**	hypofuran A	C_10_H_14_O_4_	yellow oil	—	[35]
**18**	5-(hydroxymethyl)furfural	C_6_H_6_O_3_	yellow oil	furan aldehyde	[36]
**19**	p-hydroxybenzaldehyde	C_7_H_6_O_2_	yellow oil	aromatic aldehyde	[37]
**20**	hemerocallisamine II	C_10_H_15_NO_2_	yellow oil	nitrogen-containing compound	[38]
**21**	tetrahydrofuran	C_4_H_8_O	yellow oil	furan	[39]
**22**	methyl hexadecanoate	C_17_H_34_O_2_	yellow oil	fatty acid and fatty alcohol derivative	[40]
**23**	bis(2-ethyloctyl) phthalate	C_28_H_46_O_4_	yellow oil	phthalate	[41]
**24**	(E)-2,4-dimethylhept-2-ene-1,5-diol	C_9_H_18_O_2_	yellow oil	—	[42]
**25**	5-(hydroxymethyl)tetrahydrofuran-2-carbaldehyde	C_6_H_10_O_3_	yellow oil	furan aldehyde	[43]

**Table 3:** 1H (600 MHz) and 13C (600 MHz) NMR data of 1 and 2 in CDCl3 (d in ppm, *J* in Hz).

Pos.	1		Pos.	2	
	δC, Type	δH (*J* in Hz)		δC, Type	δH (*J* in Hz)
1	49.4, CH	2.63, dd (10.8, 6.0)	1	49.4, CH	2.74, dd (11.1, 6.0)
2	35.5, CH	2.46, m;	2	35.5, CH	2.46, m;
3	132.3, CH	5.67, ddd (10.2, 4.5, 2.2)	3	132.3, CH	5.68, ddd (10.2, 4.5, 2.0)
4	125.6, CH	5.99, br.d (9.8)	4	125, CH	6.00, br.d (10.0)
5	49.9, CH	1.73, m	5	50.0, CH	1.75, m
6	73.2, CH	3.32, td (9.5, 4.3)	6	73.1, CH	3.33, td (10.0, 4.4)
7	36.3, CH_2_	2.05, m; 1.30, m	7	36.2, CH_2_	2.05, m; 1.31, m
8	24.1, CH_2_	1.79, m; 1.44, m	8	24.0, CH_2_	1.78, m; 1.45, m
9	29.5, CH_2_	1.68, m; 0.91, m	9	29.4, CH_2_	1.60, m; 0.88, m
10	34.0, CH	1.70, m	10	34.2, CH	1.73, m
11	163.7, C		11	168.3, C	
12	100.4, CH	5.64, s	12	96.3, CH	6.08, s
13	95.6, C		13	166.5, C	
14	160.1, C		14	103.9, C	
15	171.8, C		15	165.3, C	
16	17.7, CH_3_	1.00, d (7.2)	16	17.6, CH_3_	0.98, d (7.2)
17	191.7, CH		17	54.7, CH_2_	4.55, s
O-Me			18	56.5, CH_3_	3.91, s

**Table 4:** 1H (600 MHz) and 13C (600 MHz) NMR data of 3 in CDCl3 (d in ppm, *J* in Hz).

Pos.	δC, Type	δH (*J* in Hz)	Pos.	δC, Type	δH (*J* in Hz)
1	68.9, CH_2_	4.05, m	1′	174.7, C	
		3.82, dd (10.8, 3.6)	2′	72.1, CH	4.49, d (5.4)
2	53.5, CH	3.95, m	3′	126.7, CH	5.40, dd (15.0, 5.8)
3	69.8, CH	3.37, m	4′	134.3, CH	5.79, dt (14.8, 7.0)
4	134.5, CH	5.38, dd (15.0, 7.2)	5′	32.8, CH_2_	1.96, m
5	128.9, CH	5.68, dd (15.0, 5.6)	6′	29.8, CH_2_	1.22, m
6	32.6, CH_2_	1.96, m	7′	29.5, CH_2_	1.28, m
7	27.8, CH_2_	1.97, m	8′	14.2, CH_3_	0.85, t (7.0)
8	123.3, CH_2_	5.06, br. s	1″	102.9, CH	4.33, d (7.7)
9	136.0, C		2″	73.0, CH	3.25, m
10	39.9, CH_2_	1.91, t (6.0)	3″	76.1, CH	3.44, ma
11 to 14	22.8–30.0, CH_2_	1.22–1.31, m	4″	73.4, CH	3.30, ma
15	32.0, CH_2_	1.22, m	5″	76.1, CH	3.36, ma
16	22.8, CH_2_	1.24, m	6″	61.1, CH_2_	3.83, d (11.2)
17	14.2, CH_3_	0.85 (7.0)			3.70, dd (12.0, 4.2)
18	16.0, CH_3_	1.54, s	NH		7.47, d (9.1)

## Data Availability

The original contributions presented in this study are included in the article/Supplementary Materials. Further inquiries can be directed to the corresponding authors.

## Supplementary Materials

The following supporting information can be downloaded at https://www.mdpi.com/article/10.3390/molecules31213855/s1, Table S1: The gene sequences for all fungal strains.
