# Supplementary material for: Three New Secondary Metabolites from Colletotrichum boninense, an Endophytic Fungus in Polygonum multiflorum
Source: Molecules. 2026 Oct 26;31(21):3855. doi: 10.3390/molecules31213855 (PMC13764884; doi:10.3390/molecules31213855)
Supplement: Supplementary file 1 [file molecules-31-03855-s001.zip › molecules-4605027-supplementary.pdf]

Table S1 The gene sequences for all fungal strains.

Strain NO.	Gene Order	Identity (%)
H1	CGGCGAGTGAAGCGGCAA-----CAGCTCAAATTTGAAATCTGGCTCTTTTAGAGTC----CGAGTTGTAATT<br>TGCAGAGG-GCGCTTTGGCGTTGGCAGCGGTCCAA-----GTTCTTTGGAACAGGACGTCACAGAGG-GTGAGA<br>ATC--CCGTACGTGGTCGCTAGCCTTCGCCGTGTAAAGCCCCTTCGACGAGTCGAGTTGTTTGGGAATGCAG<br>CT--CTAAATGGGAGGTAAATTTCTT---CTAAAGCTAAATACTGGCCAGAGACCGATAGCGCACAAGTAGAG<br>TGATCGAAAGATGAAAAGCACTTTGGAAAGA----GAGTCAAATAGCACGTGAAAT-TGTTGAAAGGGAAGC<br>GCTTGCAGCCAGACTTGCCTGTAGTTGCTTATCCGGACTTTTGTCCGGTGCACTCTTCTGCGGGCAGGCCAG<br>CA-TCAGTCTAGGCGGTTGGATAAAGGTCTCTGT-CATGTACCTCTCTTCGGGGAGGCCTTATAGGGGAGAC<br>GACATGCAACCAGCCTGGACTGAGGTCCGCGCATCTGCTAGGATGCTGGCGTAATGGCTGTAAGCGGCCC<br>GTCTTGAAACACGGACCAAGGAGTCTAACATCTATGCGAGTGTTTGGGTGTCAAGCCCAGACGCGTAATG<br>AAAGTGAACGGAGGTGGGAACCCTTTGGGTGCACCATCGACCGATCCTGATGTCTTCGGATGGATTTGAGT<br>AAGAGCATGGCTGTTGGGACCCGAAAGATGGTGAACTATGCTTGAATAGGGTGAAGCCAGAGGAAACTCT<br>GGTGGAGGCTCGCAGCGGTTCTGACGTGCAAATCGATCGTCAAATTTGGGCATAGGGGCGAAAGACTAAT<br>CGAACTATCTAGTA-GCTGGTTCCTGCCGAAGATCCCCTCCAAGGAAACCAAAC	99.55%
H2	TAGTAACGGCGAGTGAAGCGGCAA-----CAGCTCAAATTTGAAATCTGGCTCTTTTAGAGTC----CGAGT<br>TGTAATTTGCAGAGG-GCGCTTTGGCGTTGGCAGCGGTCCAA-----GTTCTTTGGAACAGGACGTCACAGAGG<br>-GTGAGAATC--CCGTACGTGGTCGCTAGCCTTCGCCGTGTAAAGCCCCTTCGACGAGTCGAGTTGTTTGGGA<br>ATGCAGCT--CTAAATGGGAGGTAAATTTCTT---CTAAAGCTAAATACTGGCCAGAGACCGATAGCGCACAA<br>GTAGAGTGATCGAAAGATGAAAAGCACTTTGGAAAGA----GAGTCAAATAGCACGTGAAAT-TGTTGAAAG<br>GGAAGCGCTTGCAGCCAGACTTGCCTGTAGTTGCTTATCCGGACTTTTGTCCGGT-GCACTCTTCTGCGGGC<br>AGGCCAGCATCAGTCTAGGCGGTTGGATAAAGGTCTCTGT-CATGTACCTCTCTTCGGGGAGGCCTTATAG<br>GGGAGACGACATGCAACCAGCCTGGACTGAGGTCCGCGCATCTGCTAGGATGCTGGCGTAATGGCTGTAA<br>GCGGCCCGTCTTGAAACACGGACCAAGGAGTCTAACATCTATGCGAGTGTTTGGGTGTCAAGCCCAGACG<br>CGTAATGAAAGTGAACGGAGGTGGGAACCCTTTGGGTGCACCATCGACCGATCCTGATGTCTTCGGATGG<br>ATTTGAGTAAGAGCATGGCTGTTGGGACCCGAAAGATGGTGAACTATGCTTGAATAGGGTGAAGCCAGAG<br>GAAACTCTGGTGGAGGCTCGCAGCGGTTCTGACGTGCAAATCGATCGTCAAATTTGGGCATAGGGGCGAA<br>AGACTAATCGAACTATCTAGTAGCTGGTTCCTGCCGAGTCTCC CCTCCCCAGAGAAAACAAAC	99.66%
H3	GAGTGAAGCGGCA--ACAGCTCAAATTTGAAATCTGG--TCCTTGCGGCCCGAGTTGTAATTTGCAGAGG<br>ATGCTTTTGGCGCGGTGCCTTCCAAGTTCCCTGGAACGGGACGCCTTAGAGGGTGAGAGCCCCGTACGGTT<br>GGACACCAAGCCTTTGTAAAGCT----CCTTCGACGAGTCGAGTAGTTTGGGAATGCTGCTCTAA-ATGGGAG<br>GTATATTTCTT---CTAAAGCTAAATACCGGCCAGAGACCGATAGCGCACAAGTAGAGTGATCGAAAGATGA<br>AA--AGCACTTTGAAAAG-AGGGTTAAACAGCACGTGAAATTGTTAAAAGGGAAGCGCTTGTGACCAGACTT<br>GCGCCCGGTGAATCACCCAGCTCTCGCGGCTGGGGCACTTTGCCGGCTCAG--GCCAGCATCAGCTTGCCGT<br>CGGGGACAAAAGCTTCGGGAACGTAGCTCTCTTCGGGGAGTGTTATAGCCCGTTGCACAATACCCTTCGGC<br>GGGCTGAGGTACGCGCTCCGCAAGGATGCTGGCATAATGGTCATCAGCGACCCGTCTTGAAACACGGACC<br>AAGGAGTCAACCTTATGTGCGAGTGTTCGGGTGTCAAACCCCTACGCGTAATGAAAGTGAACGCAGGTGA<br>GAGCTTCGGCGCATCATCGACCGATCCTGATGTTCTCGGATGGATTTGAGTAAGAGCATATAGGGTTGGAC<br>CCGAAAGAAGGTGAACTATGCGTGTATAGGGTGAAGCCAGAGGAAACTCTGGTGGAGGCTCGCAGCGGTT<br>CTGACGTGCAAATCGATCGTCAAATATGCGCATGGGGGGCGAAAGACTAATCGAACCTTCTAGTAGCTGG<br>TTTCCGCCGAAGAT-----	99.0%
H4	CTCAGTAACGGCGAGTGAAGCGGCAACAGCTCAAATTTGAAATCTGGTC-CTTGCGGCCCGAGTTGTA<br>ATTTGCAGAGGATGCTTTTGGCGCGGTGCCTTCCAAGTTCCCTGGAACGGGACGCCTTAGAGGGTGAGAGC<br>CCCGTACGGTTGGACACCAAGCCTTTGTAAAGCT----CCTTCGACGAGTCGAGTAGTTTGGGAATGCTGCTC<br>TAA-ATGGGAGGTATATTTCTT---CTAAAGCTAAATACCGGCCAGAGACCGATAGCGCACAAGTAGAGTGA<br>TCGAAAGATGAAA--AGCACTTTGAAAAG-AGGGTTAAACAGCACGTGAAATTGTTAAAAGGGAAGCGCTT<br>GTGACCAGACTTGCGCCCGGTGAATCACCCAGCTCTCGCGGCTGGGGCACTTTGCCGGCTCAG--GCCAGCA<br>TCAGCTTGCCGTCGGGGACAAAAGCTTCGGGAACGTAGCTCTCTTCGGGGAGTGTTATAGCCCGTTGCACA<br>ATACCCTTCGGCGGGCTGAGGTACGCGCTCCGCAAGGATGCTGGCATAATGGTCATCAGCGACCCGTCTTG<br>AAACACGGACCAAGGAGTCAACCTTATGTGCGAGTGTTCGGGTGTCAAACCCCTACGCGTAATGAAAGTG<br>AACGCAGGTGAGAGCTTCGGCGCATCATCGACCGATCCTGATGTTCTCGGATGGATTTGAGTAAGAGCATA<br>TAGGGTTGGACCCGAAAGAAGGTGAACTATGCGTGTATAGGGTGAAGCCAGAGGAAACTCTGGTGGAGGC<br>TCGCAGCGGTTCTGACGTGCAAATCGATCGTCAAATATGCGCATGGGGGCGAAAGACTAATCGAACCTTCT<br>AGTAGCTGGTTTCCGCCGAAGATCCCCCCCTCAGGAAAACAAC	100.0%
H5	GATCCGGAGTAGACAACAGGGATTGCCCCAGTAACGGCGAGTGAAGCGGCAACAGCTCAAATTTGAA<br>ATCTGGCCCCAGGCCCGAGTTGTAATTTGCAGAGGATGCTTTTGGCGCG-GTGCCTTCCGAGTTCCCTGGAA<br>CGGGACGCCATAGAGGGTGAG--AGCCCCGTACGGTTGGACACCAAGCCTTTGTAAAGCTCCTTCGACGAG<br>TCGAGTAGTTTGGGAATGCTGCTCTAAATGGGAGGTACATTTCTTCTAAAGCTAAATATTGGCCAGAGACC<br>GATAGCGCACAAGTAGAGTGATCGAAAGATGAAAAGCACTTTGAAAAGAGGGTTAAACAGCACGTGAAA<br>TTGTTAAAAGGGAAGCGCTTGTGACCAGACT----TGCGTCCGGTGAATCACCCAGCTCTCGCGGCTGGGGCA<br>CTTCGCCGGCACAGGCCAGCATCAGCTTG--CCGTCGGGG-ACAAAAGCTTCAGGAACGTGGCTCCTCTCGG<br>GGAGTGTTATAGCCTGTTGTATAATACCCTTCGGCGGGCTGAGGTACGCGCTCCGCAAGGATGCTGGCGTA<br>ATGGTCATCAGCGACCCGTC--TTGAAACACGGACCAA-GGAGTCAACCTTATGTGCGAGTGTTTGGGTGTT<br>AAACC-CCTACGCGTAATGAAAGTGA-ACGCAGGTGAGAGCTTCGGCGCATCATCGACCGATCCTGATGTT<br>C--TCGGATGGATTTGAGTAAGAGCATATAGGGTTGGACCCGAAAGAAGGTGAACTATGCGTGTGTAGGGT<br>GAAGCCAGAGGAAACTCTGGTGGAGGCTCGCAGC-GGTTCTGACGTGCAAATCGAT--CGTCAAACATGCGC<br>ATGGGGGGCGAAAGACTAATCGAACCTTCTAGTAGCTGGTTTCCGCCGAAGTTTCTCCCCCTAAGGAACAA<br>CTG	99.89%
H6	AGGGATTGCCTCAGTAACGGCGAGTGAAGCGGCAACAGCTCAAATTTGAAATCTGG---C<br>CCTAGGCCCGAGTTGTAATTTGCAGAGGATGCTTTTGGTGAGGTGCCTTCCAAGTTCCCTAGAACGGG<br>ACGCCAGAGAGGGTGAGAGCCCCGTACAGTTGGACACCAAGCCTTTGTAAAGCT----CCTTCGACGAGTCG<br>AGTAGTTTGG-----GAATGCTGCTCAAAATGGGAGGTATATTTCTTCTAAAGCTAAATACCGGCCAGAGACC<br>GATAGCGCACAAGTAGAGTGATCGAAAGATGAAAAGCACTTTGA-AAAGAGGGTTAAACAGCACG---TGA<br>AATTGTTAAAAGGGAAGCGCTTGTGACCAGACTTGCGTCCGGTGAATCACCCAGCTCTCGC---GGCTGGGG<br>CACTTCGCCGGCTCAGGCCAGCATCAGCTCGCTGTCGGGGACAAAAGCTTCAGGAACGTAGCTCTCTTCGG<br>GGAGTGTTATAGCCTGTTGCATAATACCCTTCGGCGGGCTGAGGTACGCGCTCCG-CAAGGATGCTGGCAT<br>AATGGTCATCAGCGACCCGTCTTGAAACACGGACCAAGGAGTCAACCTTATGTGCG-AGTGTTCGGGTGTC<br>AAACCCCTACGCGTAATGAAAGTGAACGCAGGTGAGAGCTTCGGCGCATCATCGACCGATCCTGATGTTCT<br>CGGATGGATTTGAGTAAGAGCATATAGGGTTGGACCCGAAAGAAGGTGAACTATGCGTGTGTAGGGTGAA<br>GCCAGAGGAAACTCTGGTGGAGGCTCGCAGCGGTTCTGACGTGCAAATCGATCGTCAAACATGCGCATGG<br>GGGCGAAAGACTAATCGAACCTTCTAGTA-GCTGGTTTCCGCCGAAGTTCCCCTCAAGGAAA	99.54
H7	AGTAACGGCGAGTGAAGCGGCAACAGCTCAAATTTGAAATCTGGCCCCAGGCCCGAGTTGTAATTTG<br>CAGAGGATGCTTTTGGCGCG-GTGCCTTCCGAGTTCCCTGGAACGGGACGCCATAGAGGGTGAG--AGCCCC<br>GTACGGTTGGACACCAAGCCTTTGTAAAGCTCCTTCGACGAGTCGAGTAGTTTGGGAATGCTGCTCTAAAT<br>GGGAGGTACATTTCTTCTAAAGCTAAATATTGGCCAGAGACCGATAGCGCACAAGTAGAGTGATCGAAAG	99.76%

	ATGAAAAGCACTTTGAAAAGAGGGTTAAACAGCACGTGAAATTGTTAAAAGGGAAGCGCTTGTGACCAGA<br>CT----TGCGTCCGGTGAATCACCCAGCTCTCGCGGCTGGGGCACTTCGCCGGCACAGGCCAGCATCAGCTTG<br>--CCGTCGGGG-ACAAAAGCTTCAGGAACGTGGCTCCTCTCGGGGAGTGTTATAGCCTGTTGTATAATACCC<br>TTCGGCGGGCTGAGGTACGCGCTCCGCAAGGATGCTGGCGTAATGGTCATCAGCGACCCGTC--TTGAAAC<br>ACGGACCAA-GGAGTCAACCTTATGTGCGAGTGTTTGGGTGTTAAACC-CCTACGCGTAATGAAAGTGA-AC<br>GCAGGTGAGAGCTTCGGCGCATCATCGACCGATCCTGATGTTC--TCGGATGGATTTGAGTAAGAGCATATA<br>GGGTTGGACCCGAAAGAACGTGAACTATGCGTGTGTAGGGTGAAGCCAGAGGAAACTCTGGTGGAGGCTC<br>GCAGC-GGTTCTGACGTGCAAATCGATCGTCAAACATGCGCA-----TGGGGGCGAAGACTAATCGAACCTTC<br>TAGTAGCTGGTTTCCGCCGAGACTCCCCCTCAAGGAGAACAACC	
H8	CGGCGAGTGAAGCGGCAACAGCTCAAATTTGAAATCTGGCCCCAGGCCCGAGTTGTAATTTGCAGAG<br>GATGCTTTTGGCGCG-GTGCCTTCCGAGTTCCCTGGAACGGGACGCCATAGAGGGTGAG--AGCCCCGTACG<br>GTTGGACACCAAGCCTTTGTAAAGCTCCTTCGACGAGTCGAGTAGTTTGGGAATGCTGCTCTAAATGGGAG<br>GTACATTTCTTCTAAAGCTAAATATTGGCCAGAGACCGATAGCGCACAAGTAGAGTGATCGAAAGATGAA<br>AAGCACTTTGAAAAGAGGGTTAAACAGCACGTGAAATTGTTAAAAGGGAAGCGCTTGTGACCAGACT----T<br>GCGTCCGGTGAATCACCCAGCTCTCGCGGCTGGGGCACTTCGCCGGCACAGGCCAGCATCAGCTTG--CCGT<br>CGGGG-ACAAAAGCTTCAGGAACGTGGCTCCTCTCGGGGAGTGTTATAGCCTGTTGTATAATACCCTTCGG<br>CGGGCTGAGGTACGCGCTCCGCAAGGATGCTGGCGTAATGGTCATCAGCGACCCGTC--TTGAAACACGGA<br>CCAA-GGAGTCAACCTTATGTGCGAGTGTTTGGGTGTTAAACC-CCTACGCGTAATGAAAGTGA-ACGCAGG<br>TGAGAGCTTCGGCGCATCATCGACCGATCCTGATGTTC--TCGGATGGATTTGAGTAAGAGCATATAGGGTT<br>GGACCCGAAAGAAGGTGAACTATGCGTGTGTAGGGTGAAGCCAGAGGAAACTCTGGTGGAGGCTCGCAG<br>C-GGTTCTGACGTGCAAATCGAT--CGTCAAACATGCGCATGGGGGGCGAAAGACTAATCGA------------	99.88%
H9	AACGGCGAGTGAAGCGGCAACAGCTCAAATTTGAAATCTGGCCCCAGGCCCGAGTTGTAATTTGCAGAGG<br>ATGCTTTTGGCGCG-GTGCCTTCCGAGTTCCCTGGAACGGGACGCCATAGAGGGTGAG--AGCCCCGTACGG<br>TTGGACACCAAGCCTTTGTAAAGCTCCTTCGACGAGTCGAGTAGTTTGGGAATGCTGCTCTAAATGGGAGG<br>TACATTTCTTCTAAAGCTAAATATTGGCCAGAGACCGATAGCGCACAAGTAGAGTGATCGAAAGATGAAA<br>AGCACTTTGAAAAGAGGGTTAAACAGCACGTGAAATTGTTAAAAGGGAAGCGCTTGTGACCAGACT----TG<br>CGTCCGGTGAATCACCCAGCTCTCGCGGCTGGGGCACTTCGCCGGCACAGGCCAGCATCAGCTTG--CCGTC<br>GGGG-ACAAAAGCTTCAGGAACGTGGCTCCTCTCGGGGAGTGTTATAGCCTGTTGCATAATACCCTTCGGC<br>GGGCTGAGGTACGCGCTCCGCAAGGATGCTGGCGTAATGGTCATCAGCGACCCGTC--TTGAAACACGGAC<br>CAA-GGAGTCAACCTTATGTGCGAGTGTTTGGGTGTTAAACC-CCTACGCGTAATGAAAGTGA-ACGCAGGT<br>GAGAGCTTCGGCGCATCATCGACCGATCCTGATGTTC--TCGGATGGATTTGAGTAAGAGCATATAGGGTTG<br>GACCCGAAAGAAGGTGAACTATGCGTGTGTAGGGTGAAGCCAGAGGAAACTCTGGTGGAGGCTCGCAGC-<br>GGTTCTGACGTGCAAATCGAT--CGTCAAACATGCGCATGGGGGGCGAAAGACTAATCGAACCTTCTAGTA<br>GCTGGTTTCCGCCGAAGTTTCCCTCAGGGAAAAAAA	99.88%
H10	AACGGCGAGTGAAGCGGCAACAGCTCAAATTTGAAATCTGGCCCCAGGCCCGAGTTGTAATTTGCAGAGG<br>ATGCTTTTGGCGCG-GTGCCTTCCGAGTTCCCTGGAACGGGACGCCATAGAGGGTGAG--AGCCCCGTACGG<br>TTGGACACCAAGTCTTTGTAAAGCTCCTTCGACGAGTCGAGTAGTTTGGGAATGCTGCTCAAAATGGGAGG<br>TATATTTCTTCTAAAGCTAAATATTGGCCAGAGACCGATAGCGCACAAGTAGAGTGATCGAAAGATGAAA<br>AGCACTTTGAAAAGAGGGTTAAACAGCACGTGAAATTGTTAAAAGGGAAGCGCTTATGACCAGACT----TG<br>CGCCCGGTGAATCACCCAGCTCTCGCGGCTGGGGCACTTTGCCG---GCTCAGGCCAGCATCAGCTCGCTGT<br>CGGGGACAAAAGCTTCGGGAACGTAGCTCTCTTCGGGGAGTGTTATAGCCCGTTGCACAATACCCTTCGGC<br>GGGCTGAGGTACGCGCTCCGCAAGGATGCTGGCGTAATGGTCATCAGCGACCCGTC--TTGAAACACGGAC<br>CAA-GGAGTCAACCTTATGTGCGAGTGTTTGGGTGTCAAACCCCTACGCGTAATGAAAGTGAACGCA--GGT<br>GAGAGCTTCGGCGCATCATCGACCGATCCTGATGTTC--TCGGATGGATTTGAGTAAGAGCATATAGGGTTG<br>GACCCGAAAGAAGGTGAACTATGCGTGTATAGGGTGAAGCCAGAGGAAACTCTGGTGGAGGCTCGCAGC-<br>GGTTCTGACGTGCAAATCGAT--CGTCAAATATGCGCATGGGGGCGAAAGACTAATCGAACCTTCTAGTAG<br>CTGGTTTCCGCCGAAGTT--CTCCCCCTCAAGAAAAACCAC	100%
H11	TATCCGGGAGAGACAACAGGGATTGCCTCAGTAACGGCGAGTGAAGCGGCAACAGCTCAAATTTGAAATC<br>TGG---CCCTAGGCCCGAGTTGTAATTTGCAGAGGATGCTTTTGGTGAGGTGCCTTCCAAGTTCCCTAGAACG<br>GGACGCCAGAGAGGGTGAGAGCCCCGTACAGTTGGACACCAAGCCTTTGTAAAGCT----CCTTCGACGAGT<br>CGAGTAGTTTGG-----GAATGCTGCTCAAAATGGGAGGTATATTTCTTCTAAAGCTAAATACCGGCCAGAGA<br>CCGATAGCGCACAAGTAGAGTGATCGAAAGATGAAAAGCACTTTGA-AAAGAGGGTTAAACAGCACG---T<br>GAAATTGTTAAAAGGGAAGCGCTTGTGACCAGACTTGCGTCCGGTGAATCACCCAGCTCTCGC---GGCTGG<br>GGCACTTCGCCGGCTCAGGCCAGCATCAGCTCGCTGTCGGGGACAAAAGCTTCAGGAACGTAGCTCTCTTC<br>GGGGAGTGTTATAGCCTGTTGCATAATACCCTTCGGCGGGCTGAGGTACGCGCTCCG-CAAGGATGCTGGC<br>ATAATGGTCATCAGCGACCCGTCTTGAAACACGGACCAAGGAGTCAACCTTATGTGCG-AGTGTTCGGGTG<br>TCAAACCCCTACGCGTAATGAAAGTGAACGCAGGTGAGAGCTTCGGCGCATCATCGACCGATCCTGATGTT<br>CTCGGATGGATTTGAGTAAGAGCATATAGGGTTGGACCCGAAAGAAGGTGAACTATGCGTGTGTAGGGTG<br>AAGCCAGAGGACACTCTGGTGGAGGCTCGCAGCGGTTCTGACGTGCAAATCGATCGTCAAACATGCGCAT<br>GGGGGCGAAAGACTAATCGAACCTTCTAGTAGCTGGTTTCC-GCCGAAGTTCCCCCCTCAGGGAT	99.43%
H12	CTAGTAACGGCGAGTGAAGCGGCAA-----CAGCTCAAATTTGAAATCTGGCTCTTTTAGAGTC----CGAG<br>TTAATTT------GCAGAGGGCGCTTTGGCTTTGGCAGCGGTCCAAGTTCCTTGGAACAGGACGTCACAGAGG-<br>GTGAGAATC--CCGTACGTGGTCGCTGGCTA-TTGCCGTGTAAAGCCCCTTCGACGAGTCGAGTTGTTTGGGA<br>ATGCAGCT--CTAAATGGGAGGTACATTTCTT---CTAAAGCTAAATATTGGCCAGAGACCGATAGCGCACAA<br>GTAGAGTGATCGAAAGATGAAAAGCACTTTGGAAAGAGAGTCAAACAGCAC----GTGAAAT-TGTTGAAAG<br>GGAAGCGCTTGCAGCCAGACTTGCTTACAGTTGCTCATCCGGGTTTCTACCCGGTGCACTCTTCTGTAGGC<br>AGGCCAGCA-TCAGTTTGGGCGGTAGGATAAAGGTCTCTGT-CACGTACCTCCTTTCGGGGAGGCCTTATAG<br>GGGAGACGACATACTACCAGCCTGGACTGAGGTCCGCGCATCTGCTAGGATGCTGGCGTAATGGCTGTAA<br>GCGGCCCGTCTTGAAACACGGACCAAGGAGTCTAACATCTATGCGAGTGTTTGGGTGTCAAGCCCGAGCG<br>CGTAATGAAAGTGAACGGAGGTGGGAACCCGCAAGGGTGCACCATCGACCGATCCTGATGTCTTCGGAAG<br>GATTTGAGTAAGAGCATGGCTGTTGGGACCCGAAAGATGGTGAACTATGCTTGA-ATAGGGTGAAGCCAGA<br>GGAAACTCTGGTGGAGGCTCGCAGCGGTTCTGACGTGCAAATCGATCGTCAAATTTGGGCATAGGGGCGA<br>AAGACTAATCGAACTATCTAGTAGCTGGTTCCTGCCGAAGTTCCCCCTCAAGGAA	99.89%
H13	AGAAGTTGGGGGTTTAACGGCAGGGCACCGCCAGGGCCTTCCAAAGCGAGATATAACTACTACGCTC<br>GGGGTCCTGGCGAGCTCGCCACTGAATTTCAGGGCCTGCTCCTCTCGAAGCAGTGCCCCATCACCAAGCCA<br>GGCTTGAGGGTTGAAATGACGCTCGAACAGGCATGCCCCCCGGAATACCAGGGGGCGCAATGTGCGTTCA<br>AAGATTCGATGATTCACTGAATTCTGCAATTCACATTACTTATCGCATTTCGCTGCGTTCTTCATCGATGCC<br>AGAACCAAGAGATCCGTTGTTGAAAGTTTTGATTCATTTATGTTTTTTACTCAGAGATTCACTATAGAAACA<br>AGAGTTTA<br>GTTGGCCGCCGGCGGGCTGCTCCCCGTCTCCGGGGGGCCCCAGTGAAGGGGCCGGCCTGACGCCGAG<br>GCAACAGTAGGTATAAGTTCACAAAGGGTTTCTGGGTGCGCCGAAGCGCGTTCCAGCAATGATCCCTCCGC<br>TGGTTCACCAACGGAGACCTTGTTACGACTTTTACTTC	99.44%
H14	GAGTGAAGCGGCAACAGCTCAAATTTGAAATCTGGCCTTCGGGTCCGAGTTGTA------ATTTGTAGAGG<br>ATGCTTTTGGTGAGGTGCCTTCCGAGTTCCCTGGAACGGGACGCCATAGAGGGTGAGAGCCCCGTCTGGTT<br>GGACACCGATCCTCTGTAAAGCTCCTTCGACGAGTCGAGTAGTTTGGGAATGCTGCTCTAAATGGGAGGTA<br>TATGTCTTCTAAAGCTAAAT--ATTGGCCAGAGACCGATAGCGCACAAGTAGAGTGATCGAAAGATGAAAA<br>GCACTTTGAAAAGAGAGTTAAAAAGTACGTGAAATTGTTGAAAGGGAAGCGCTCATGACCAGACTTGGGC<br>TT-GGTTGATCATCCAGGGTTCTCCCTGGTGCACTCTTCCAGTTCAGGCCAGCATCAGTTCGCTGTGGGGGA<br>TAAAGGCTTTGGGAATGTGGCTCCC-TCGGGAGTGTTATAGCCCATTGTGTAATACCCTG--TGGTGGACTGA<br>GGTTCGCGCATTCGCAAGGATGCTGGCGTAATGGTCATCAGCGACCCGTCTTGAAACACGGACCAAGGAG-<br>---TCGTCTTCGTATGCGAGTGTTCGGGTGTTAAACCCCTACGCGTAATGAAAGTGAACGCAGGTGAGAGCT<br>TCGGCGCATCATCGACCG-ATCCTGATGTTCTCGGATGGATTTGAGTAAGAGCATACGGGGCCGGACCCGA	99.53%

	A--AGAA	
	GGTGAACTATGCCTGTATAGGGTGAAGCCAGAGGAAACTCTGGTGGAGGCTCGCAGCGGTTCTGACG<br>TGCAAATCGATCGTCAAATATGGGCATGGGGGGCGAAAGACTAATCGAACCTTCTAGTAGCTGGTTTCCGC<br>CGAAGATCTCCCTCTAAGGGATCCA	
H15	GAGTGAAGCGGCAACAGCTCAAATTTGAAATCTGGCCTTCGGGTCCGAGTTGTAATTTGTAGAGGATG<br>CTTTTGGTGAGGTGCCTTCCGAGTTCCCTGGAACGGGACGCCATAGAGGGTGAGAGCCCCGTCTGGTTGGA<br>CACCGATCCTCTGTAAAGCTCCTTCGACGAGTCGAGTAGTTTGGGAATGCTGCTCTAAATGGGAGGTATAT<br>GTCTTCTAAAGCTAAAT--ATTGGCCAGAGACCGATAGCGCACAAGTAGAGTGATCGAAAGATGAAAAGCA<br>CTTTGAAAAGAGAGTTAAAAAGTACGTGAAATTGTTGAAAGGGAAGCGCTCATGACCAGACTTGGGCTT-G<br>GTTGATCATCCAGGGTTCTCCCTGGTGCACTCTTCCAGTTCAGGCCAGCATCAGTTCGCTGTGGGGGATAA<br>AGGCTTTGGGAATGTGGCTCCC-TCGGGAGTGTTATAGCCCATTGTGTAATACCCTG--TGGTGGACTGAGGT<br>TCGCGCATTCGCAAGGATGCTGGCGTAATGGTCATCAGCGACCCGTCTTGAAACACGGACCAAGGAG----T<br>CGTCTTCGTATGCGAGTGTTCGGGTGTTAAACCCCTACGCGTAATGAAAGTGAACGCAGGTGAGAGCTTCG<br>GCGCATCATCGACCG-ATCCTGATGTTCTCGGATGGATTTGAGTAAGAGCATACGGGGCCGG-ACCCGAAA<br>GAAAGGTGAACTATGCCTGTATAGGGTGAAGCCAGAGGAAACTCTGGTGGAGGCTCGCAGCGGTTCTGAC<br>GTGCAAATCGATCGTCAAATATGGGCATGGGGGGCGAAAGACTAATCGAACCTTCTAGTAGCTGGTTTCC<br>GCCGAGACTCCCCTCAGAGGAAAAACAAAACC------------	99.88%
H16	TATCGGGGAGCAGACCAACAGGGATTGCCCCAGTAACGGCGAGTGAAGCGGCAACAGCTCAAATTTG<br>AAATCTGGCCCCAGGCCCGAGTTGTAATTTGCAGAGGATGCTTTTGGCGCG-GTGCCTTCCGAGTTCCCTGG<br>AACGGGACGCCATAGAGGGTGAG--AGCCCCGTACGGTTGGACACCAAGCCTTTGTAAAGCTCCTTCGACG<br>AGTCGAGTAGTTTGGGAATGCTGCTCAAAATGGGAGGTATATTTCTTCTAAAGCTAAATATAGGCCAGAGA<br>CCGATAGCGCACAAGTAGAGTGATCGAAAGATGAAAAGCACTTTGAAAAGAGGGTTAAACAGCACGTGA<br>AATTGTTAAAAGGGAAGCGCTTGTGACCAGACT----TGCGTCCGGTGAATCACCCAGCTCTCGCGGCTGGGG<br>CACTTCGCCGGCACAGGCCAGCATCAGCTTG--CCGTCGGGG-ACAAAAGCTTCAGGAACGTAGCTCTCTTC<br>GGGGAGTGTTATAGCCTGTTGCATAATACCCTTCGGCGGGCTGAGGTACGCGCTCCGCAAGGATGCTGGCG<br>TAATGGTCATCAGCGACCCGTC--TTGAAACACGGACCAA-GGAGTCAACCTTATGTGCGAGTGTTTGGGTG<br>TTAAACCCCTACGCGTAATGAAAGTGAACGCA--GGTGAGAGCTTCGGCGCATCATCGACCGATCCTGATGT<br>TC--TCGGATGGATTTGAGTAAGAGCATATAGGGTTGGACCCGAAAGAAGGTGAACTATGCGTGTGTAGGG<br>TGAAGCCAGAGGAAACTCTGGTGGAGGCTCGCAGC-GGTTCTGACGTGCAAATCGAT--CGTCAAACATGCG<br>CATGGGGGCGAAAGACTAATCGAA--CCTTCTAGTAGCTGGTTTCCGC-CGAAGATCCCTTTTCAAGGAAAA<br>ACA	100%
